# ImmunoPET: Antibody-Based PET Imaging in Solid Tumors

**DOI:** 10.3389/fmed.2022.916693

**Published:** 2022-06-28

**Authors:** Reyhaneh Manafi-Farid, Bahar Ataeinia, Shaghayegh Ranjbar, Zahra Jamshidi Araghi, Mohammad Mobin Moradi, Christian Pirich, Mohsen Beheshti

**Affiliations:** ^1^Research Center for Nuclear Medicine, Shariati Hospital, Tehran University of Medical Sciences, Tehran, Iran; ^2^Department of Radiology, Martinos Center for Biomedical Imaging, Massachusetts General Hospital and Harvard Medical School, Boston, MA, United States; ^3^Division of Molecular Imaging and Theranostics, Department of Nuclear Medicine, University Hospital Salzburg, Paracelsus Medical University, Salzburg, Austria; ^4^Rajaie Cardiovascular Medical and Research Center, Iran University of Medical Sciences, Tehran, Iran

**Keywords:** immunoPET, monoclonal antibody (mAb), molecular imaging, solid tumors, immunoimaging, PET

## Abstract

Immuno-positron emission tomography (immunoPET) is a molecular imaging modality combining the high sensitivity of PET with the specific targeting ability of monoclonal antibodies. Various radioimmunotracers have been successfully developed to target a broad spectrum of molecules expressed by malignant cells or tumor microenvironments. Only a few are translated into clinical studies and barely into clinical practices. Some drawbacks include slow radioimmunotracer kinetics, high physiologic uptake in lymphoid organs, and heterogeneous activity in tumoral lesions. Measures are taken to overcome the disadvantages, and new tracers are being developed. In this review, we aim to mention the fundamental components of immunoPET imaging, explore the groundbreaking success achieved using this new technique, and review different radioimmunotracers employed in various solid tumors to elaborate on this relatively new imaging modality.

## Introduction

There is an expanding insight into the role of different molecules and pathways in the development and progression of cancer. The growing knowledge about the involved molecules and processes has resulted in the modification in cancer management; therefore, targeted therapies and immunotherapies are increasingly utilized to treat different malignancies (1–3). This process was accelerated by the production of monoclonal antibodies (mAbs), following advances in DNA technology and Ab engineering (4). The targets can be membrane receptors, enzymes, or various molecules in signaling pathways, which are overexpressed or specifically present in a particular tumor or its microenvironment (5). Targeting molecules include Abs and Ab fragments, small molecule inhibitors, selective high-affinity ligands, some peptides, and aptamers (5, 6).

The first human radioimmunoimaging was conducted in 1978 using ^131^l-labeled whole immunoglobin G (lgG) targeting carcinoembryonic antigen (CEA) (7) with inherent drawbacks. Since then, significant efforts have been implemented to develop ideal radioimmunoimaging tracers and radiopharmaceuticals for different cancers. The ideal tracer should be target-specific, biologically inert, highly stable in serum, minimally immunogenic, with rapid biodistribution and background clearance. Physiochemical characteristics to facilitate radiolabeling are also crucial (6, 8). For instance, manufactured small-sized Ab fragments (Fab) show higher specificity and rapid biodistribution and provide superior imaging characteristics over whole Abs (9, 10).

In the era of ever-growing targeted therapy, there is a requirement for accurate targeted imaging. Although immunohistochemistry (IHC) is the integral modality for detecting biomarkers (11), the non-invasive evaluation of the whole-body remains a compelling field of research, especially for patient selection and response evaluation. Medical imaging has a fundamental role in managing solid tumors, among which positron emission tomography (PET) is of particular importance (12). PET-based imaging demonstrates different functional and biochemical procedures occurring in normal tissues and malignant tumors at the cellular and molecular levels (12, 13).

The recent advances in PET acquisition systems, providing highly sensitive imaging (13), coupled with developments in labeling methods (14) and the specific targeting offered by mAbs, build the foundation of immunoPET. ImmunoPET is molecular imaging used for (1) the evaluation of biodistribution of Abs or their fragments in normal and malignant tissues, (2) the non-invasive detection of expression of target molecules and their heterogeneity in whole-body, and 3) prediction of response to targeted therapies (15). Although the concept of immunoPET is simple, it is an umbrella term covering almost all aspects of medical imaging, including oncology, infection/inflammation, neurological diseases, and drug development.

In this review, we aimed to provide a simplified summary of the current state of immunoPET in oncology. First, we briefly present the principles of immunoPET. Afterward, we focus on the Ab-based immunoPET in solid tumors and discuss the various developed probes in preclinical and clinical studies for each cancer.

## The Concept of ImmunoPET

PET is a non-invasive and powerful imaging procedure with a wide range of clinical and research applications. PET provides the three-dimensional mapping of organs and lesions using a radioactive tracer. Radionuclides are incorporated either into compounds normally used by the organs, such as glucose, or into molecules that bind to receptors, peptides, cytokines, or other components of cellular pathways (16, 17). Recent advances in the development of PET systems and sophisticated software enable rapid, highly sensitive imaging (18). The combination of the superior targeting specificity of immune system-associated molecules and the inherent high sensitivity of PET technique establishes the principle of ImmunoPET (19). These tracers can specifically target various molecular pathways involved in the tumor biology (4).

Generally, the successful development of immunoPET in oncology is highly dependent on knowledge about the processes involved in tumor biology, choice of tumor-targeting vectors, radionuclides and chelators, and conjugation strategies. A vast number of molecules and processes are involved in tumor development and progression (20), the details of which are beyond the scope of this review. Some of the studied targets for imaging are discussed in more detail in the next section.

A variety of tumor-targeting vectors have been investigated for immunoPET. Full-length Abs are among the most used forms (21). Abs and associated amino acid-based macromolecules have been developed to display high specificity and binding affinity toward molecular targets overexpressed by cancer cells and tumor microenvironment (4). Their development dates back to the beginning of the twentieth century when Paul Ehrlich brought up the “magic bullet” idea to seek out and eradicate the spirochete of syphilis without affecting normal tissues (22).

MAbs are Abs with specificity for one particular epitope on an antigen (23). Despite the clinical success, these Abs come with a number of limitations including slow blood clearance, serum sickness, low target-to-background ratio (TBR), and the necessity of repetitive imaging (24). Moreover, high costs of production limit their use in developing countries. Thus, smaller Ab constructs have been engineered to overcome these limitations (25). Engineered Abs have a faster clearance rate, higher TBR, and are suggested to penetrate solid tumors more effectively (26).

Immunoglobulin G (IgG) is the most common type of Abs. IgG is composed of two main parts, crystallizable fragment (Fc) and antigen-binding Fragment (Fab), which contain polypeptides of heavy and light chains forming the constant and variable fragments of the IgG. The smaller fragments used for radiolabeling contain the variable domain. Fab and (Fab′)2 fragments are made by omitting the Fc region from Abs. They can have more rapid renal clearance and improved tumor penetration. However, their production is difficult and cannot be obtained from all subclasses of Abs (27). The smaller molecule, single-chain fragment variable (scFv), consists only of light and heavy variable chains. scFvs are produced more easily and are one of the most popular used fragments (28). The chains are attached with a non-covalent association, making scFv normally unstable. A disulfide bond between chains is used to increase stability, forming disulfide fragment variable (dsFv), and single-chain disulfide fragment variable (scdsFv). Proper tumor uptake and retention could be achieved by increasing the valency of scFvs molecules. Multivalent scFvs such as diabodies, tribodies and tetrabodies are favorable agents for radioimmunoimaging. In comparison with monovalent scFvs, tumor the retention time is augmented in multivalent scFvs. Also, their clearance time is shorter than whole Abs but equivalent to monovalent scFvs (25). scFvs are cleared through the urinary system, and there is significant retention in the kidneys (29).

**Figure 1:**
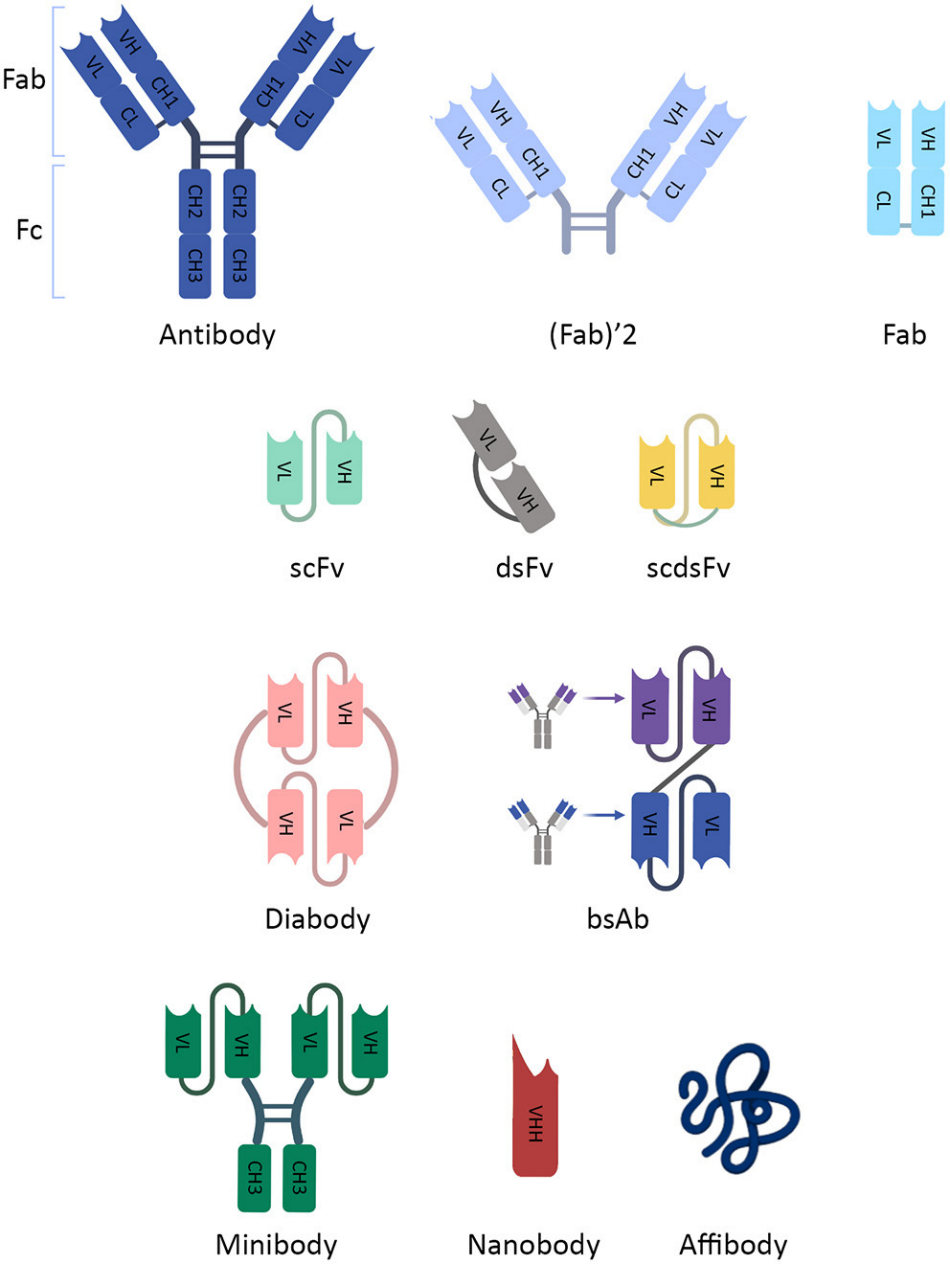
The schematic illustration of an antibody and its fragments. The schematic illustration of an antibody and its fragments. IgG has two main parts, crystallizable Fragment (Fc) and antigen-binding Fragment (Fab). They contain fragments (constant [C] and variable [V]) which are composed of polypeptides of heavy (H) and light (L) chains. Due to inappropriate imaging characteristics, smaller fragments of Abs, which contain the variable domain for targeting, were developed. Fab and (Fab′)2 fragments are made by omitting the Fc region from Abs. scFv consists only of light and heavy variable chains. dsFv has a disulfide bond between chains, and scdsFv has both non-covalent and disulfide bonds to increase stability. scFv dimers (diabody) and multimers (not shown) provide more than one binding site. They can be multivalent scFvs containing different targeting fragments (bsAb). Minibody is an engineered Ab fragment, a combination of scFv with human IgG1 CH3. Nanobodies are the smallest fragment produced from natural heavy-chain-only Abs. CH, constant heavy chain; CL, constant light chain; dsFv, disulfide fragment variable; Fab, antibody fragment; Fc, crystallizable fragment; IgG, Immunoglobulin G; scdsFv, single-chain disulfide fragment variable; scFv, single-chain fragment variable; VH, variable heavy chain; VL, variable light chain; VHH, variable heavy chain of heavy-chain-only antibodies.

Minibody is another engineered Ab fragment, produced by combining scFv molecule with human IgG1 constant heavy chain-3 (CH3) (30). Application of minibody is a mean to surmount slow renal clearance, which is the major problem of scFvs (29). Nanobodies or single-domain antibodies (sdAbs) are the smallest fragments of Abs obtained from the camelid heavy-chain-only Abs (31). Nanobodies are easier to produce and are more stable than scFvs (31). Finally, there is a smaller engineered targeting protein, affibody, which is derived from the IgG binding region. Affibodies seem to be a suitable protein for imaging but suffer from rapid clearance and decreased avidity to the targets (Figure 1) (27).

## ImmunoPET in Different Malignancies

ImmunoPET is known to provide excellent specificity and sensitivity in detecting some tumors (32, 33). However, some drawbacks include suboptimal imaging properties (feasibility, long imaging protocols), low expression of the targets in tumoral lesions, and high background activity in some organs.

Several molecules are involved in the development of different malignancies (Table 1). Receptor tyrosine kinases (RTKs) have been among the most explored targets for developing anticancer therapeutic and imaging agents. Substantial efforts have been made to establish immunoPET probes for revealing the heterogeneous expression of RTKs. The human epidermal growth factor (HER) family is one of the most evaluated RTKs, including four members, HER1-4 (101). A number of mAbs and Ab fragments have been developed for preclinical and clinical studies targeting anti-epidermal growth factor receptor (EGFR, known as HER1) in various malignancies (101), HER2 mostly in breast cancer (102), and HER3 in different solid tumors (103–106). Other RTKs, such as vascular endothelial-derived growth factor/receptor (VEGF/VEGFR) (107) and platelet-derived growth factor/receptor (PDGF/PDGFR) (82), and insulin-like growth factor-1 receptor (IGF-1R) (74) have also been targeted for immunoPET imaging.

**Table 1:** Biomarkers targeted in different malignancies for immunoPET imaging.

**Biomarker**	**Type**	**Role**
A33 (34)	Transmembrane glycoprotein	Interacts in cell adhesion.
ACKR3 (35)	Transmembrane protein	Interacts in cell adhesion, angiogenesis, tumor development and progression.
Axl (36)	Transmembrane RTK	Responsible for cancer development and progression Associated with survival.
CA 15-3 (MUC1) (37)	Transmembrane glycoproteins	Expressed on normal and malignant epithelial cells, possessing different functions (cell surface protection, cellular adhesion).
CA125 (MUC16) (38)	Transmembrane glycoproteins	Expressed on normal and malignant epithelial cells, possessing different functions (cell surface protection).
CA6 (39)	tumor-associated mucin 1-sialoglycotope antigen	Results of aberrant glycosylation in cancer cells.
CA-IX (40)	Cell surface protein	Overexpressed in hypoxia. Associated with tumor aggressiveness.
Cadherin-17 (CDH17) (41)	Transmembrane protein	Plays role in the adhesion of cells.
CD11b (42)	Transmembrane protein, a part of macrophage-1 antigen	Expressed on tumor-associated myeloid cells. Interacts in cell adhesion, migration, and complement 3 activation.
CD30 (43)	Transmembrane glycoprotein, member of TNF superfamily	Upregulated in T-cell activation. Regulates cytotoxic function of NK and T-cells.
CD38 (44)	A cell surface receptor and enzyme	Interacts in cell proliferation. May have role in resistance to ICIs.
CD44 (45)	A non-kinase transmembrane glycoprotein	Interacts in cell adhesion, migration, and metastasis. Responsible for cancer development and progression
CD44v6 (46)	An oncogenic variant of the cell surface molecule CD44	Responsible for cancer progression, invasion, and metastasis, overexpressed in squamous epithelium. Associated with resistance to therapy.
CD47 (47)	Transmembrane protein (macrophage immune checkpoint)	Plays role in the downregulation of immune response. Associated with poor prognosis.
CD133 (Prominin-1) (48)	Transmembrane glycoprotein	A stem cell identification marker. Associated with progression and poor prognosis.
CD146 (MUC18) (49, 50)	A cell surface protein	Interacts with VEGFR, activates epithelial-to-mesenchymal transition, which promotes metastatic potential and resistance to apoptosis. Associated with progression, invasion, and metastasis.
CD147 (51)	transmembrane protein of Ig superfamily	Inducts MMPs and VEGF expression. Associated with tumor growth and metastasis.
CDCP1 (52)	Transmembrane receptor	Regulates signaling pathways in tumors. Relays cancer promotion and associated with poor prognosis.
CEA (53–55)	Cell surface glycoprotein	Modulates intercellular adhesion, promotes cellular aggregation, and mediates transduction. Correlates with poorer survival. Enhances the potential of metastasis.
CTLA-4 (CD152) (56)	A membrane protein (Immune check point)	Plays role in the downregulation of immune response.
CXCR4 (57)	Transmembrane receptor for the chemokine CXCL12	Plays role in chemotaxis and cell proliferation.
Dll4 (58)	A ligand of Notch family transmembrane receptors	Activates Notch signaling and improves vascular function in tumors. Associated with poor prognosis.
Disease-associated ECM protein (59)	ECM proteins	ECM plays role in invasion, prognosis, angiogenesis, and resistance to therapies.
Endoglin (CD105) (60)	Accessory receptor for TGF-β	Overexpressed in endothelial of tissues with angiogenesis. Associated with poorer prognosis.
EpCAM (CD326) (61)	glycoprotein	Interacts in cell adhesion, intercellular interaction and migration.
FAP-α (62)	Transmembrane serine protease	Interacts in multiple mechanisms involved in tumor proliferation invasion, progression and resistance to therapy. Associated with poor prognosis.
Gal-3 (63)	Galactoside-binding protein	Modulates cell growth. Associated with prognosis.
GITR (64)	Co-stimulatory molecule for T-cell	Differential immune T-cell response.
GPC3 (65)	Cell-surface protein	Regulates cell growth. Associated with poor prognosis.
GRP78 (66)	A heat shock protein	Induced by lack of glucose. Associated with poor prognosis.
HER1 (EGFR) (67)	Transmembrane RTK	Involved in signal transduction, responsible for transcription of various genes. Enhances tumor cell survival, proliferation, and differentiation. Related with resistance to treatment.
HER2 (CD340) (68)	Transmembrane RTK	Involved in signal transduction, responsible for transcription of various genes. Important role in the growth, progression, and metastasis.
HER3 (69)	Transmembrane RTK	Responsible for cancer development and progression. Associated with poor prognosis and resistance to therapy.
HGF (70)	Cytokine	A ligand for MET. Associated with tumor development, progression and therapy resistance.
hk2 (71)	trypsin-like enzyme	Enhances sperm motility. Released in blood when the prostate gland structure is compromised.
ICAM-1 (CD54) (72)	Transmembrane protein	Plays role in cell adhesion.
ICOS (CD278) (73)	Co-stimulatory molecule for T-cell	Differential immune T-cell response.
IGF-1 (74)	Transmembrane RTK	Plays role in the development of cancer, proliferation, apoptosis, angiogenesis, tumor invasion, resistance to therapy
Integrin αvβ6 (75)	Cell surface receptor	Interacts with cell adhesion. Plays role in progression.
L1CAM (76)	Transmembrane protein	Plays role in cell adhesion, proliferation, migration, and invasion.
LGR5 (77)	Transmembrane glycoprotein, a marker of stem cells	Incorporates in tumor growth, therapy, and likely recurrence. Associated with metastasis, resistance, and poor prognosis.
MET (70)	Transmembrane RTK	Receptor of HGF. Tumor development, progression and therapy resistance
MG7 (78)	Gastric cancer-specific antigen	Overexpressed in gastric cancer. Associated with poor prognosis.
MSLN (79)	Membrane-bound surface glycosylphosphatidylinositol	Help tumor peritoneal implantation, proliferation and survival
MT1-MMP (80)	Endopeptidases	Degradation of ECM helping cell migration. Associated with tumor progression and metastasis.
OX40 (CD134) (64)	Co-stimulatory molecule for T-cell	Differential immune T-cell response.
PD-1/L1 (CD274) (81)	Transmembrane protein (immune checkpoint)	Plays role in the downregulation of immune response. Associated with resistance to therapy.
PDGF/PDGFR (82)	Transmembrane RTK	Incorporates in tumor cell growth and angiogenesis.
Syndecan-1 (CD138) (83)	Transmembrane cell-surface heparan sulfate proteoglycans	Affects several steps in tumor progression and facilitate metastasis. Correlates with poor prognosis and an aggressive phenotype.
Periostin (84)	ECM protein	Plays role in adhesion and motility in tumor microenvironment
peroxiredoxin-I (85)	Cell surface receptor	Plays role in oxidative stress. Prognostic factor for lung cancer.
PSA (86)	Kallikrein-like serine protease	Enhances sperm motility. Released in blood when the prostate gland.
PSCA (87)	cell surface protein	Overexpressed in prostate cancer. Plays role in signal transduction. Correlates with progression, metastasis and poor prognosis.
PSMA (88)	A transmembrane glycoprotein	Overexpressed in prostate cancer. Increases aggressiveness of prostate cancer.
RAGE (89)	Transmembrane receptor	Binding to multiple ligands. Plays role in transition to cancer.
RANKL (90)	A member of TNF	Plays role in osteoclastogenesis and bone Homeostasis. Plays resistance to immunotherapy.
TAG-72 (91)	Membrane-bound glycoprotein	A glycoprotein with mucin properties, overexpressed in some adenocarcinomas. Associated with progression.
TAM (92)	Macrophage	Associated with metastasis and poor response different therapies. Associated with poor survival.
TF (CD142) (93)	Transmembrane glycoprotein receptor	Initiates of the coagulation cascade.
TfR (94)	Transmembrane glycoprotein	Involves in iron uptake and cell growth.
TGF-β (95)	Cytokine	Plays a significant role cell proliferation, differentiation and apoptosis. Associated with poorer prognosis
TIL (96)	Lymphocyte	Responsible for malignant cells' death. The presence of TIL correlates with survival.
TIM-3 (97)	Immune checkpoint	Plays role in the downregulation of immune response.
TRA-1-60 (98)	Cell-surface antigen, a biomarker of stem cell	Associated with drug resistance and recurrence.
TROP-2 (99)	Transmembrane glycoprotein	Possesses stem-cell like qualities. Regulates proliferation, transformation and progression.
VEGF/VEGFR (100)	Transmembrane RTK	Responsible for tumor angiogenesis. Associated with progression and poor prognosis.

*ACKR3, Atypical chemokine receptor 3, known as C-X-C chemokine receptor type 7; CA 15-3, Carcinoma antigen 15-3; CA125, Carbohydrate antigen 19-9; CA6, Carbonic anhydrase 6; CA-IX, Carbonic anhydrase-IX; CD, Cluster differentiation; CDCP1, CUB domain-containing protein 1; CEA, Carcinoembryonic antigen; CTLA-4, Cytotoxic T-lymphocyte associated protein-4; CXCR4, C-X-C chemokine receptor type 4; Dll4, Delta-like ligand 4; ECM, Extracellular matrix; EGFR, Epidermal growth factor receptor; EpCAM, Epithelial cellular adhesion molecule; EphA2, Ephrin receptor A2; FAP-α, Fibroblast activation protein-alpha; Gal-3, Galactoside-binding protein galectin-3; GITR, Glucocorticoid-induced tumor necrosis factor receptor; GPC3, Glypican-3; GRP78, Glucose-regulated protein; HER, Human epithelial receptor; HGF, Hepatocyte growth factor; hk2, Human kallikrein-related peptidase 2; ICAM-1, Intercellular adhesion molecule-1; ICI, Immune checkpoint inhibitor; ICOS, Inducible T-cell costimulatory receptor; IGF-1, Insulin-like growth factor-1; LGR5, Leucine-rich repeat-containing, G protein-coupled receptor 5; MMP, Matrix metalloproteinases; MSLN, Mesothelin; MT1-MMP, Membrane type-1 matrix metalloproteinases; MUC, Mucin; OX40, Tumor necrosis factor (ligand) superfamily, member 4; PD-1/PD-L1, Programmed cell death protein-1/ligand; PDGF/PDGFR, Platelet-derived growth factor/receptor; PSA, Prostate-specific antigen; PSMA, Prostate-specific membrane antigen; RAGE, Receptor for advanced glycation end products; RANKL, Receptor activator of nuclear factor kappa B ligand; RTK, Receptor tyrosine kinase; TAG-72, Tumor-associated glycoprotein-72; TAM, Tumor-associated macrophages; TF, Tissue factor; TfR, Transferrin receptor; TGF-β, Transforming growth factor beta; TIL, Tumor-infiltrating lymphocyte; TIM-3, T-cell immunoglobulin and mucin domain-containing-3; TNF, Tumor necrosis factor; TROP-2, Known as tumor-associated calcium signal transducer 2; VEGF/VEGFR, Vascular endothelial-derived growth factor/receptor*.

Processes other than only high Ag-Ab affinity are required for appropriate targeting. The heterogeneous tracer uptake and high physiologic activity, especially in the liver and lymphoid tissue, can make imaging with these probes challenging (15, 106, 108). Preinjection of the cold tracer might decrease the hepatic uptake (109). Also, long radioimmunotracer clearance time for whole Abs can limit the tumor visualization (67, 110, 111). On the other hand, the renal retention of the smaller fragments is higher (112–115). Moreover, the poor vascular permeability of the tumors rather than the unfavorable characteristics of the probe can affect the outcome (17, 116).

The binding of programmed cell death protein ligand-1 (PD-L1) on the tumor cells to PD-1 on T-cells suppresses T-cell function and helps tumors escape the immune system. Immune checkpoint inhibitors (ICIs) are Abs that block PD-L1 and show therapeutic effect in various malignancies (117). Several radiolabeled ICIs have been developed for immunoPET, showing high physiologic uptake in lymphoid tissues (118, 119). Preinjection of unlabeled anti-PD-L1 may be useful for the PD-L1 expression evaluation (120). Also, T-cells in the tumor microenvironment impact response to therapies (121, 122) that have been evaluated in different malignancies (123–126), which can be used as a biomarker for response evaluation.

Many other proteins and molecules are overexpressed in different tumors, making them potential targets for immunoPET imaging. Some molecules and pathways are engaged in the development of various malignancies, and some are particularly found in distinct tumors. Below, we review the relevant evaluated probes in different malignancies.

### Breast Cancer

Breast cancer is a heterogeneous malignancy with different features and outcomes (127). HER2 is a well-known prognostic biomarker and an effective therapeutic target (127, 128). Despite multiple therapeutic agents (127, 129), there is an unmet need to identify the patients who may benefit from these expensive and potentially toxic pharmaceuticals (109). There is intrapatient and intratumoral heterogeneity in expression of HER2 (15, 109, 130, 131), which is also dynamic over the disease course (129). This impacts the therapy response and the treatment options for patients. In this regard, several studies investigated radiolabeled mAbs and their fragments to develop new imaging agents targeting HER2. Proper tumor uptake and visualization of HER2-positive lesions were demonstrated using ^89^Zr-labeled pertuzumab and trastuzumab in the preclinical (132, 133) and phase I clinical studies (102, 131, 134, 135).

Although new lesions were found (102), some HER2-positive lesions showed no significant uptake (102, 134), and the detection rate of 79–89% was reported (102, 134). Interestingly, HER2-targeting tracer uptake, suggesting HER2-positivity, was demonstrated in metastases from HER2-negative primary tumors (130, 131). The heterogenicity was also illustrated in metabolism on [^18^F]FluoroDeoxyGlucose PET/computed tomography ([^18^F]FDG PET/CT), with a discrepancy in the same-lesion standardized uptake value (SUV) between [^18^F]FDG and [^64^Cu]Cu-trastuzumab in HER2-positive lesions (109). This observation suggests a complementary role for both scans, further evaluated in a multicenter study (15). Gebhart et al. showed that pre-treatment [^89^Zr]Zr-trastuzumab PET/CT accompanied by early metabolic response assessment using [^18^F]FDG PET/CT best predicts the outcome after trastuzumab therapy (15). Moreover, to guide the treatment approach, [^89^Zr]Zr-trastuzumab PET/CT increased physicians' confidence or altered management in a substantial ratio of patients (136). Also, early changes in [^89^Zr]Zr-trastuzumab uptake correlated with response to therapy with a new experimental agent (137). The liver uptake is substantial with whole Abs, which may be decreased by the cold pharmaceutical preinjection (109).

To improve pharmacokinetics, scholars developed different HER2-targeting small Ab fragments in preclinical studies (138–141). Also, to enable early imaging and feasibility of labeling with more accessible radionuclides, such as ^68^Ga, promising Ab fragments are investigated in early phase clinical studies (112–115, 142), showing a high correlation between the probe uptake, [^68^Ga]Ga-ABY-025, and pathology (142). Expectedly, the renal retention is higher using Ab fragments (112–115).

Regulation of the immune system by ICIs have shown therapeutic efficacy in triple-negative breast cancer (143). Evaluating PD-L1 expression, Bensch et al. reported a heterogeneous tracer uptake in a few cancer types, including triple-negative breast cancer (144). The intensity of [^89^Zr]Zr-atezolizumab (anti-PD-L1) uptake best correlated with outcome compared to IHC in their study (144). Currently, the role of [^89^Zr]Zr-atezolizumab PET/CT is being investigated for patient selection for ICIs therapy (NCT02478099 and NCT02453984). Moreover, [^89^Zr]Zr-Avelumab, another anti-PD-L1 ligand, is developed for the imaging of breast cancer in preclinical studies (145, 146).

In addition, CEA is a cell surface adhesion molecule (53), correlating with poor survival in breast cancer (54). Imaging with anti-CEA immunoPET seems promising. TF2 is a bispecific trivalent mAb comprising two humanized anti-CEA and an antihistamine-succinyl-glycine (HSG) hapten Fab fragments. This unique structure enables TF2 to be used in pretargeted imaging of malignancies with CEA expression (147). It showed more bone metastases than conventional imaging (148, 149) and helped delineate the clinical target volume for stereotactic body radiotherapy in CEA-positive metastatic breast cancer (148).

Furthermore, markers of tumor anagenesis have been targeted for immunoPET, which can be used as response prediction biomarkers to anti-angiogenesis therapies (150). The anti-VEGF, [^89^Zr]Zr-bevacizumab, localized almost all primary breast lesions (25/26) but was limited in the detection of metastatic lymph nodes (4/10) (151). Also, the uptake of anti-endoglin (anti-cluster of differentiation [CD]105), an accessory receptor for transforming growth factor-β (TGF-β), was demonstrated in mice models (60, 152–155).

There are also other prognostic biomarkers which have been evaluated for the imaging of triple-negative breast cancer in preclinical studies, such as syndecan-1 (CD138) (83) and IGF-1 (74).

### Lung Cancer

Non-small cell lung cancer (NSCLC) is also a heterogeneous malignancy (156). Radioimmunoimaging in lung cancer has been performed in preclinical and clinical studies for various purposes.

Guidelines recommend targeted therapies for treating NSCLC using anti-EGFR and anti-VEGF/R mAbs (157). Similar to other therapies, it is crucial to predict the response before or early during the treatment. Radiolabeled Abs targeting EGFR (such as panitumumab and cetuximab) (10, 158–160) and VEGF/VEGFR (bevacizumab and ramucirumab) (150, 161, 162) have shown increased uptake in tumoral cells, including NSCLC (163–165). In pilot clinical studies, the feasibility and safety of administration of [^89^Zr]-cetuximab (111) and [^89^Zr]Zr-bevacizumab (166) were demonstrated in patients with NSCLC, showing TBRs of 0.9–4.5 and 0.7–8.6 in tumoral lesions for [^89^Zr]Zr-cetuximab and [^89^Zr]Zr-bevacizumab, respectively (111, 166).

ICIs are also increasingly administered in NSCLC patients with expression of PD-1/PD-L1 (167–169). Scholars have developed radiolabeled-ICIs to non-invasively evaluate the expression of PD-1/PD-L1 (170–172) and cytotoxic T-lymphocyte associated protein-4 (CTLA-4) (56). In clinical studies, [^89^Zr]Zr-nivolumab (anti-PD-1), [^18^F]BMS-986192 (anti-PD-L1 adnectin) (118) and [^89^Zr]Zr-pembrolizumab (anti-PD-1) (173) demonstrated heterogeneous uptake and delineation of lesions larger than 2cm in some patients. The tumor uptake was insignificantly higher in responders to the anti-PD-1 treatment (118, 173).

The presence of tumor-infiltrating lymphocytes (TILs) correlates with survival in different malignancies (96). Also, the amount of T-cell infiltration after immunotherapy in the tumor microenvironment impacts the response (121, 122). The non-invasive evaluation of these cells may predict response to immunomodulatory therapies. Probes have been developed to monitor TILs dynamics after therapy (123–126). In this regard, an anti-CD8 minibody, [^89^Zr]Zr-IAB22M2C, showed a favorable biodistribution (174), and currently, it is under investigation in phase II clinical trial for different solid tumors (NCT03802123). Also, the presence of tumor-associated macrophages (TAMs), indicating poorer survival (92), CD30, a marker of T-cell activation (175), and inducible T-cell costimulatory receptor (ICOS), a costimulatory signaling molecule (73) have been assessed using immunoPET.

MET is an RTK, a receptor of hepatocyte growth factor (HGF), which plays a role in the development, progression, and therapy resistance (70). ImmunoPET shows potential in the non-invasive evaluation of MET expression (176, 177). Another biomarker, CD146, interacts with VEGFR (17). It is overexpressed in about 50% of lung cancers and correlates with poor survival (178, 179). The [^64^Cu]Cu-YY146 mAb showed a positive correlation with CD146 expression (49) and strong binding to CD146-expressing cell lines (50). Daratumumab (anti-CD38 mAb) is approved for treating multiple myeloma and is being investigated in a phase I/II clinical trial (NCT03665155). CD38 may have a role in resistance to immunotherapies (44). [^89^Zr]Zr-daratumumab uptake has been reported in the CD38-expressing lung cancer model (44). Other probes against RTK [HER2 (180) and Axl (36)] and peroxiredoxin-I, a marker of oxidative stress (85), have also been assessed in NSCLC.

### Colorectal Cancer

Tumor biomarkers are being investigated in metastatic colorectal cancer and are used to guide patient selection due to variable responses to available targeted therapies (181, 182).

Anti-EGFR therapy is used for Kirsten rat sarcoma virus (KRAS) wild-type tumors; however, not all patients respond to this therapy (183, 184). Anti-EGFR immunoPET using cetuximab and panitumumab showed specific but heterogeneous uptake in EGFR-expressing preclinical models (67, 160, 185, 186) and colorectal cancer patients (184). Evaluating the clinical impact in patient selection for therapy, van-Helden et al. failed to demonstrate a relation between [^89^Zr]Zr-cetuximab-positivity and treatment response or outcome (183). Also, the high liver sequestration of [^89^Zr]Zr-cetuximab in normal liver tissue is vital in colorectal cancer, limiting its diagnostic ability in liver metastasis (108).

Anti-CEA Ab scintigraphy was used for the first human radioimmunoimaging (7), and it is a relevant target for immunoPET (55). Different mAb and small Ab fragments have been developed for preclinical studies (187–189). However, pretargeted imaging using bispecific Ab (bsAb) seems more promising. Bispecific tracers (anti-CEA × anti-HSG) demonstrated highly specific tumor localization (190–193) and may outperform [^18^F]FDG PET/CT (194). A recent phase I trial depicted the safety and feasibility of [^68^Ga]Ga-IMP288 PET/CT (an anti-CEA × anti-HSG) with TF2 pretargeting, showing encouraging diagnostic performance for the detection of colorectal cancer metastases with the sensitivity of 88% and specificity of 100% (195).

T-cell redirecting bsAb are novel agents that target different antigens on T-cells and tumor cells, facilitating T-cell antitumor response (196). Using bsAbs, an anti-CEA × anti-CD3 [[^89^Zr]Zr-AMG 211 (197)], and an anti-CEA × anti-interleukin-2 [[^89^Zr]Zr-CEA-IL2v (198)] showed specific uptake in CEA-positive tumors, highlighting the potential value of such probes in immunomodulatory therapy response monitoring.

A33 is a glycoprotein responsible for cell adhesion, overexpressed in 95% of colorectal cancers (34). [^124^I]I-huA33 PET/CT localized tumoral lesions in colorectal cancer patients (34, 199); however, the tracer uptake was also seen in normal bowel (34). To improve pharmacokinetics, pretargeted imaging was also evaluated, producing high-quality images in the preclinical studies (200, 201). The expression of another prognostic cell adhesion glycoprotein, CD44, was also demonstrated in colorectal cancer models (202, 203). Tumor-associated glycoprotein-72 (TAG-72) was also targeted (204), which is a prognostic cell membrane molecule overexpressed in colorectal cancer with mucin (MUC) properties.

Moreover, the cancer stem cell hypothesis suggests the possible role of cancer stem cells in the progression of malignancies. In this regard, a few molecules have successfully targeted stem cells in preclinical studies, such as CD133 (205) and LGR5 (leucine-rich repeat-containing, G protein-coupled receptor 5) (77, 206). Additionally, probes monitoring TILs in response to immunotherapy have been evaluated in colorectal cancer models (124, 125).

There are other factors involved in tumor development, progression, and poor prognosis in colorectal cancer, assessed as targets for immunoPET in preclinical studies for the detection of colorectal cancer cell lines, including HER3 (69), angiogenesis factors [VEGFR (161) and PDGFRβ (82)], and hypoxia (M75 targeting carbonic anhydrase-IX [CA-IX]) (40, 207).

### Prostate Cancer

Ongoing studies are striving to improve imaging in prostate cancer. The prostate-specific membrane antigen (PSMA) is known as a suitable target for prostate cancer imaging and therapy (208, 209). Agents targeting PSMA are classified into three groups: Abs, aptamers, and PSMA inhibitors (210). Here, we discuss Abs against the PSMA molecule and other targets for immunoPET imaging of prostate cancer.

ProstaScint ([^111^In]In-capromab-pentitide) was the first FDA-approved mAb-based imaging, employing a mAb against PSMA (7E11) to detect occult pelvic lymph node metastases and recurrence of prostate cancer (211, 212). However, the binding of 7E11 to the intracellular epitope of PSMA resulted in low sensitivity for detecting metastases (213). To overcome this limitation, extracellular-binding mAbs were developed, such as J591 (88), labeled with imaging and therapeutic radionuclides (214–216). In a clinical study, the humanized [^89^Zr]Zr-J591 (huJ591) showed accuracies of 60% and 95% for the detection of soft-tissue and bone metastases (217). However, mAbs have a prolonged circulation time due to their large size (218). In this regard, the third generation mAbs (minibodies/diabodies) were developed (219, 220). [^89^Zr]Zr-IAB2M, a minibody, showed a favorable biodistribution (221) and promising diagnostic performance compared to magnetic resonance imaging (MRI) and [^68^Ga]Ga-PSMA PET/CT (222). It had a sensitivity of 88% and a low specificity of 34% (222). Also, other anti-PSMA Abs, [^64^Cu]Cu-3/A12 (223, 224), and [^124^I]I-ScFvD2B (225), have successfully localized PSMA-positive prostate cancer xenografts.

One of the challenges in prostate cancer imaging is tumors with no or negligible PSMA uptake. Therefore, tracers targeting biomarkers of poorer prognosis may help detect or treat this subset of patients.

Various other Abs or Ab fragments have been evaluated in preclinical studies. Prostate stem cell antigen (PSCA) is a cell membrane protein that plays a part in signal transduction. Because of the slow kinetics of whole mAbs, different Ab fragments have been produced to target PSCA in preclinical studies (87, 226–228). It may become helpful in response evaluation to anti-androgen therapy (87). Low non-specific uptake has been reported with [^124^I]I-A11 minibody (226) and slightly better with its smaller fragment, [^89^Zr]Zr-A2cDb diabody (227, 229). Furthermore, TROP-2, known as tumor-associated calcium signal transducer 2, is a glycoprotein possessing stem-cell like qualities, overexpressed in some malignancies, (99). Pretargeted imaging with bsAbs, anti-TROP-2 × anti-HSG, may improve TBR and provide a fast, sensitive, and specific tool for prostate cancer imaging (218, 230). Also, a scFv against CD133, [^89^Zr]Zr-HA10, localized in aggressive prostate cancer models (231, 232).

Others are anti-EpCAM Ab against epithelial cell adhesion molecule (233), [^64^Cu]Cu-1A2G11 targeting IGF-1R (234), [^89^Zr]Zr-Bstrongomab against TRA-1-60, an stem cell biomarker (98), [^89^Zr]Zr-11B6 against androgen receptor-regulated human kallikrein-related peptidase 2 (71), and [^89^Zr]Zr-5A10 against free prostate-specific antigen (235).

Prostate cancer cells have been targeted with different Abs and Ab fragments; however, few have been investigated in human studies. Mainly because PSMA inhibitors, showing favorable theranostic values (208, 209), have cast a shadow on other targets in prostate cancer. However, immunoPET may further help evaluate the aggressive variants (231, 232) or androgen receptor status or guide androgen receptor deprivation therapy (71). Also, it can have a role in radioimmunotherapy of patients who are not candidates or not responding to the currently available therapies.

### Gastric Cancer

ImmunoPET has been investigated in gastric cancer only in limited studies. Targeted therapy against HER2 is recommended to treat gastric cancer with HER2-overexpression (168). ImmunoPET has shown promising results in the non-invasive evaluation of HER-positive lesions. Radiolabeled anti-HER2 (236, 237) and anti-HER3 (mAb3481) (238) successfully detected gastric cancer cells in mice models. Also, to assess therapy response after HER2-targeted therapy, a decrease in [^89^Zr]Zr-trastuzumab uptake was demonstrated in the gastric cancer xenografts (239). Additionally, the dynamics of HER2 expression after pretreatment with lovastatin was evaluated using HER2 targeted immunoPET (240, 241). In initial clinical studies, [^89^Zr]Zr-trastuzumab has shown a wide range of no to intense uptake in HER2-positive gastroesophageal cancers (242, 243). The uniformly high [^89^Zr]Zr-trastuzumab uptake in all lesions was associated with a good response to therapy (243).

A few studies evaluated the MET/HGF pathway in gastric cancer. Radiolabeled rilotumumab (anti-HGF mAb) and onartuzumab (anti-MET mAb) non-invasively detected HGF and MET expression, respectively (244, 245). Additionally, MG7, a gastric cancer-specific antigen, is a prognostic biomarker (78). PET imaging of anti-MG7 Abs in gastric cancer xenografts showed a favorable binding affinity (246), but its application may be limited due to the overexpression of MG7 in helicobacter-related gastric disease (247). Also, radiolabeled anti-cadherin17 Ab (CDH17, an adhesion protein) was introduced as a potential probe for CDH17-positive gastric cancer (248).

### Hepatocellular Carcinoma (HCC)

Imaging HCC, especially for lesions smaller than 2cm, is still challenging (249). There are only few studies that used immunoPET to evaluate HCC. Glypican-3 (GPC3) is a cell surface protein overexpressed in HCC (65). Different anti-GPC3 ^89^Zr-labeled mAbs have been introduced, showing specific uptake in xenografts (250–252). To overcome the long biological half-life and weak tumor penetration, a F(ab′)2 fragment ([^89^Zr]Zr-αGPC3-F(ab')2) was also developed (253). CD146 and CD38 are also expressed in HCCs. Imaging with an anti-CD146 dual-labeled tracer (^89^Zr- and near-infrared fluorophore (NIRF), YY146-ZW8000), and an anti-CD38 ([^64^Cu]Cu-daratumumab) showed specific tracer uptake in preclinical studies (254, 255).

### Esophageal Cancer

ImmunoPET of esophageal cancer using multiple targets can provide useful information to detect primary tumors and metastases and identify tumor phenotype to facilitate patient selection for targeted therapies (57, 84, 256, 257).

Anti-EGFR imaging has been evaluated in a few preclinical studies, showing potential for [^64^Cu]Cu-cetuximab PET/CT for the EGFR-expression detection and patient selection for cetuximab treatment (257, 258). Moreover, the detection of HER2 expression was evaluated in a small number of patients with gastroesophageal cancer (242). Similar to other malignancies, there was a wide range of uptake intensity from no uptake to SUV of 22.7 (242). Additionally, the decrease in vascular density after anti-VGEF treatment was depicted by [^89^Zr]Zr-bevacizumab immunoPET (259). Finally, the overexpression of some biomarkers, such as periostin, an extracellular matrix (ECM) protein (84), and atypical chemokine receptor 3 (ACKR3), a cell adhesion transmembrane protein (35), was non-invasively depicted in esophageal cell lines.

### Head and Neck Squamous Cell Carcinoma (HNSCC)

A few targets such as EGFR and CD44v6 have been evaluated in HNSCC (260, 261). Therapeutic Abs targeting EGFR showed promising outcomes in HNSCC treatment (262). Hence, ImmunoPET can be a valuable approach for diagnosis, treatment response prediction, and RIT planning in this group of cancers (46, 263–266). Several studies assessed the correlation of radiolabeled cetuximab uptake with EGFR expression to predict response to anti-EGFR therapies in HNSCC (67, 266–268), showing a clear tumor visualization in almost all patients (8/9) in phase I clinical trial (111). Similar to other malignancies, a mismatch of [^89^Zr]Zr-cetuximab (67, 111, 268) and [^64^Cu]Cu-panitumumab (116) uptake and EGFR expression was reported. However, an anti-EGFR affibody strongly correlated with EGFR expression alteration in response to cetuximab (269). Heat shock protein 90 (HSP90) is significantly associated with many oncoproteins and an interesting target for therapy (260). After HSP90 inhibitor therapy in a preclinical model, the amount of [^124^I]I-cetuximab uptake was decreased in xenografts, but the uptake of [^124^I]I-CD4v6 was unaffected (260). CD44v6 is a specific isomer of CD44, expressed in cells with squamous differentiation and overexpressed in squamous cell carcinomas in different organs (270). Preclinical studies on engineered anti-CD44v6 Abs, Ab fragments, and minibodies reported specific tumor uptake and more favorable kinetics in HNSCC animal models (46, 271, 272). In one of the first clinical studies,^89^Zr-labeled anti-CD44v6, U36, detected primary tumors in all subjects and had equal performance to CT and MRI for the metastatic lymph node detection (273).

### Thyroid Cancer

Thyroid cancer is a common malignancy, effectively managed with radioactive iodine. However, management of less common thyroid cancer subtypes is challenging. Recently, various molecules were evaluated for targeted imaging and therapy in these cancers (274, 275).

Expression of PDGFRα has been correlated with treatment resistance and risk of recurrence in papillary thyroid carcinoma (PTC). [^64^Cu]Cu-D13C6, a PDGFRα targeting mAb, had specific uptake in PTC models, which can be of significant potential given that PTC is the most prevalent type of thyroid cancer (276). Moreover, β-galactoside-binding protein galectin-3 (Gal-3) is a biomarker, expressed only in thyroid cancer cells (63). ^89^Zr-labeled targeting IgG and Fab fragments successfully detected thyroid cancer lesions and can potentially be used for identifying recurrence and metastasis (277–281).

Some anaplastic thyroid carcinomas (ATC) overexpress HER2. In this regard, dual-tracer imaging by ^89^Zr- and IRDye 800CW-labeled pertuzumab depicted HER2-expressing ATC cell lines (282). Similar results were observed for an intercellular adhesion molecule-1 (ICAM-1), and tissue factor (TF or CD142, a mediator of hemostasis and inflammation) targeting probes in ATC models (72, 283). These findings can be of significant value in the diagnosis and management of this aggressive subtype. [^124^I]I-U36, an anti-CD44v6, also demonstrated high ATC tumor accumulation independent of the iodine uptake (284).

Medullary thyroid carcinoma (MTC) is a rare subtype, significantly expressing CEA. The first clinical trial of pretargeted PET with TF2, a bispecific trivalent mAb of anti-CEA × anti-HSG, in relapsing MTC patients showed high tumor uptake (285). Moreover, the TF2 probe had superior sensitivity over [^18^F]fluoro-l-dopa ([^18^F]FDOPA) PET/CT (148, 286) and CT (286) in detecting metastatic lesions.

### Pancreatic Cancer

Pancreatic ductal adenocarcinoma (PDAC) is the most common pancreatic malignancy (52). There is a need to improve the imaging modalities for the diagnosis and staging of PDAC (287, 288). Various Ab-based targets have been developed and are majorly evaluated only in preclinical studies, which may promote the role of immunoPET in PDAC (288).

Carbohydrate antigen 19-9 (CA 19-9), the known serum marker for PDAC, is detected on tumor cells in the vast majority of patients (289). Among agents targeting CA 19-9, a mAb, 5B1, demonstrated very high affinity and specificity (290), and immunoPET with [^89^Zr]Zr-5B1 showed remarkable radiotracer uptake in the CA 19-9-positive models (291). Also, a dual-labeled tracer (^89^Zr- and NIRF) successfully delineated tumoral lesions (292). It showed negligible non-specific uptake in CA 19-9-negative tumoral cell lines (292). However, the circulating CA 19-9 and long mAb half-life are problematic, increasing the background activity. In this regard, preinjection of cold 5B1 improved the image quality (293). Additionally, other pretargeting methods were assessed to overcome the drawbacks, including the development of a new-generation tracer (which exploits the advantages of reagents) (294) and an Ab-gold nanoparticle conjugate (295).

In the first clinical trial, [^89^Zr]Zr-HuMab-5B1 PET/CT successfully detected the primary sites of PDACs, metastases, and small lymph nodes, overlooked by other imaging techniques (296). The tumor uptake was high, showing an average SUV of 19.7 ± 2.9, and the pretargeting increased TBR (296). Interestingly, serum CA 19-9 levels did not affect the physiologic distribution (296). Another ongoing phase I trial is assessing the performance of [^89^Zr]Zr-HuMab-5B1 PET/CT in CA 19-9-positive tumors (NCT02687230).

Imaging with mAb was first conducted in PDAC using a murine Ab targeting MUC (297). Mucins are transmembrane glycoproteins highly expressed on several epithelial cancers, usually with an altered glycosylation pattern (37, 298). MUC1 or cancer antigen 15-3 (CA 15-3) may have a role in the management of PDAC (299). Also, carbohydrate antigen 125 (CA125), an extracellular domain of MUC16, is a well-known ovarian cancer biomarker (38). The newer generation of Abs targeted the unshed domain of MUC16 rather than CA125 and showed specific tracer uptake in PDAC xenografts with superior imaging characteristics (38).

Integrin αvβ6 is a cell surface protein, overexpression of which correlates with tumor progression (300) and seems as a potential theranostic agent in PDAC (301). However, the αvβ6-targeting peptides (302–304) are superior to Abs (300) and are more investigated.

Tissue factor (TF) is a transmembrane protein that plays a role in initiating coagulation and regulation of inflammation. The radiolabeled anti-TF, ALT-836, localized PDAC cell lines (93, 305). Additionally, a bsAb consisting of anti-TF × anti-endoglin improved binding affinity and localization of the xenografts (306, 307).

ECM proteins play a significant role in invasion, prognosis, angiogenesis, and resistance to therapies in some malignancies (59). Matrix metalloproteinases (MMPs) degrade ECM, helping cell migration. Abs targeting an ECM (59), a MMP protein (80), and a MMP inducer (CD147) (51) have localized PDAC in mice models.

Mesothelin (MSLN) is a membrane-bound glycoprotein overexpressed in more than 80–100% of pancreatic and ovarian cancer with an unknown specific role in tumor progression (108). MSLN-based immunoPET was previously used to evaluate the efficacy of MSLN-targeted antibody-drug conjugate (ADC) in preclinical models (308). In a clinical study on a small group of ovarian and pancreatic cancer patients, an ^89^Zr-labeled anti-MSLN Ab detected the majority of malignant lesions with minimal non-specific uptake. Tumor tracer uptake also correlated with MSLN expression on IHC, but no correlation was observed with progression-free survival (309).

Indeed, many other probes have been assessed in only animal models, targeting RTKs [IGF-1R (310) and EGFR (311, 312)], PD-L1 (120), PSCA [A11 minibody (313) and a dual-tracer with ^124^I and NIRF-A2cDb-800 (229)], TROP-2 (314), CEA (315, 316), ICAM-1 (317), and MET (318), as well as other less-evaluated radioimmunotracer against TRA-1-60 (98), CUB domain-containing protein 1 (CDCP1)(52), receptor for advanced glycation end products (RAGE) (319), glucose-regulated protein 78 (GRP78) (66), and transferrin receptor (TfR) (94).

### Renal Cell Carcinoma (RCC)

CA-IX is a highly expressed membrane-bound antigen in RCC. Girentuximab is the chimeric version of G250, a mAb targeting CA-IX (320). Tumor uptake of radiolabeled G250 was established in preclinical and early clinical studies (321–324).

[^124^I]I-girentuximab showed sensitivity and specificity of 86%, outperforming CT with 76% and 47%, respectively, in the REDECT clinical trial. However, performance declined for masses smaller than 2 cm (32, 33). ^89^Zr revealed more favorable characteristics and substituted ^124^I in further studies (325–327). [^89^Zr]Zr-girentuximab PET/CT altered clinical management in various inconclusive diagnostic scenarios (328). Adding [^89^Zr]Zr-girentuximab and [^18^F]FDG PET/CT scans to CT improved lesion detection in metastatic RCC (329). High baseline SUV on [^89^Zr]Zr-girentuximab also correlated with longer time to progression (330). Ongoing clinical trials will further elaborate on the diagnostic accuracy and theranostic potential of radiolabeled girentuximab (320, 331).

Furthermore, in preclinical studies, [^89^Zr]Zr-atezolizumab localized PD-L1-positive RCC (332), and [^64^Cu]Cu-bevacizumab reflected treatment response to everolimus, a mammalian target of rapamycin (mTOR) inhibitor (333). This finding was also observed in a clinical trial on everolimus in metastatic RCC; however, further clinical studies are required to confirm these findings (334).

### Melanoma

The first trials of Ab-based imaging in melanoma date back to more than 25 years ago when Abs against the melanin-associated antigen (335) and a mouse monoclonal anti-melanoma Ab (336) were used for imaging in patients with metastatic malignant melanoma.

Currently, targeted therapies are the main systemic treatments for advanced melanoma (337, 338). It is crucial to document the presence of targets before the initiation of therapy to achieve the best response and avoid side effects (339, 340). Several preclinical studies showed favorable binding affinity and imaging of melanoma cell lines using Abs and Ab fragments targeting immune checkpoints, PD-1/PD-L1 (9, 119, 340–345) and CTLA-4 (346). However, the uptake in the lymphoid tissue was also high (119, 347). Moreover, radiotherapy-induced PD-L1 upregulation was demonstrated in melanoma mouse models, using radiolabeled anti-PD-L1 Abs (348, 349). Another immune checkpoint, T-cell immunoglobulin and mucin domain-containing-3 (TIM-3), was a successful target for murine melanoma models (97). One clinical study evaluated [^89^Zr]Zr-pembrolizumab in melanoma, reporting relations between tracer uptake and response to therapy and survival in patients with advanced or metastatic disease (347). Also, an ongoing phase II clinical trial is investigating the biodistribution and tumor uptake of [^89^Zr]Zr-ipilimumab (anti-CTLA-4) in advanced melanoma (NCT03313323).

In addition to immune checkpoints, the number of CD4- and CD8-positive T-cells (121, 122) and the presence of costimulatory signaling molecules (64), namely OX40 (member 4 of tumor necrosis factor [TNF] family) and GITR (glucocorticoid-induced TNF Receptor), in the tumor microenvironment impact the response to immunotherapy. Preclinical studies using immunoPET probes successfully detected CD4- and CD8-positive rich tissues and upregulation of costimulatory substances (64, 122, 174, 350, 351). These probes have the potential to monitor immune response following different therapies. Moreover, immunoPET targeting ICAM-1 (72), CD146 (352), VEGF (353), and integrin-αvβ6 (75) was also successful in the detection of melanoma cells in preclinical studies.

### Ovarian Cancer

Ovarian cancer comprises multiple subtypes and a diverse profile of overexpressed molecules (354). Moreover, the lack of effective specific treatment makes such molecules valuable for targeted therapies guided by immunoPET (108).

With the wide use of anti-HER2 mAbs, several studies evaluated ^89^Zr/^64^Cu-labeled anti-HER2 mAbs and their engineered fragments in preclinical models and compared different chelators and labeling strategies, or their optimal performance in vivo (138, 140, 355–361). Radiolabeled-trastuzumab has been used to monitor response not only to anti-HER2 mAbs but also to HSP90 inhibitors, which downregulate HER2 (361, 362). Using dual labeled mAbs combines pre-operative PET imaging and intra-operative optical imaging data. It can improve precise tumor and metastasis excision, especially in metastasis-prone tumors, such as ovarian cancer. Dual-labeled pertuzumab with ^89^Zr and NIRF was used successfully for image-guided tumor resection in ovarian cancer xenografts (363).

Furthermore, VEGF imaging can be a response prediction biomarker to anti-angiogenesis therapies (150). [^89^Zr]Zr-bevacizumab was a sensitive marker of early response to HSP90, mTOR, and VEGF inhibitors in ovarian cancer models (364–366).

CA125, an extracellular domain of MUC16, is a serological biomarker for treatment monitoring and recurrence of ovarian cancer (298). Preclinical studies on the ^89^Zr/^64^Cu-labeled oregovomab, an anti-CA125 mAb, reported high uptake in ovarian cancer xenografts (367, 368). Furthermore, [^89^Zr]Zr-oregovomab detected histologically-confirmed lymph node involvement (368). The newer generation of Abs targeting MUC16 showed superior imaging characteristics (38, 369). Additionally, an ^89^Zr-labeled anti-MUC1 or CA 15-3 also showed proper performance in vivo (37). The first clinical study on a Fab targeting carbonic anhydrase 6 (CA6) epitope of MUC1 reported the safety of this probe in an ovarian cancer patient, and the probe correctly reflected the low tumor expression of CA6 observed in IHC (370).

REGN4018, a T-cell engaging bsAb targeting MUC16 × CD3, is currently being investigated in a clinical trial for ovarian cancer (NCT03564340). It had high specific uptake in tumors and lymphoid organs in preclinical studies (371). [^89^Zr]Zr-ERY974, a bsAb targeting CD3ε on T-cells × GPC3, showed high specific uptake in GPC3-positive ovarian cancer xenografts ingrafted with immune cells compared to xenografts in immunodeficient mice, highlighting the potential value of such probes in immunotherapy response monitoring (372).

Finally, as mentioned in the pancreatic cancer section, MSLN expression may be non-invasively evaluated in ovarian cancer, using ^89^Zr-labeled anti-MSLN Ab (309), which may help patient selection for therapy.

### Central Nervous System (CNS)

Precision imaging and targeted therapy in the central nervous system (CNS) is challenging due to the limited distribution of radiopharmaceuticals beyond the blood-brain barrier (BBB), especially for high molecular weight compounds such as Abs. However, disruption of BBB by tumors can lead to the appropriate probe uptake (373). Nanobodies are attractive probes for CNS malignancies given their small size, easy BBB penetration, and faster blood clearance (374). ImmunoPET has also been helpful to monitor brain drug delivery alteration in response to different methods of increasing BBB permeability (375–378) and predict response to intrathecal radioimmunotherapy (379).

Several studies, mainly preclinical, investigated radioimmunotracers targeting molecules involved in angiogenesis, such as VEGF (380, 381), CD146 (382, 383), and PDGFRβ (384) in the setting of brain tumors. On clinical studies of the mentioned targets, [^89^Zr]Zr-bevacizumab imaging was feasible and safe in children with diffuse intrinsic pontine glioma; however, significant uptake heterogeneity was observed (385). Interestingly, high expression of PSMA by neovascular endothelium has been reported in a number of highly vascularized tumors, including gliomas and some brain metastases (386). This phenomenon makes PSMA a potential target to monitor response to anti-angiogenesis treatments in brain malignancies. Matsuda et al. showed high PSMA expression in histological specimens for a wide range of brain malignancies, including gliomas and metastatic brain lesions. Next, they successfully imaged three patients with recurrent glioma or brain metastasis with [^89^Zr]Zr-IAB2M (387).

Additionally, the TGF-β is a known cytokine involved in the development of various malignancies (95). A radioimmunotracer targeting TGF-β, [^89^Zr]Zr-fresolimumab, was successfully produced (388). In a clinical trial on high-grade glioma patients undergoing anti-TGF-β treatment, [^89^Zr]Zr-fresolimumab accumulated in most tumors, except for some lesions smaller than 10 mm or those with radionecrosis (95).

Another group of glioma tracers evaluates immune system components in the tumor microenvironment by targeting immune cell infiltration and activation markers, such as CD8+ lymphocytes (389), its co-stimulatory molecule, OX40 (390), a marker of tumor-associated myeloid cells, CD11b (391, 392), a phagocytosis checkpoint molecule, CD47 (393), and lymphocyte checkpoint molecule/ligand, PD-1/PD-L1 (378, 394).

Finally, immunoPET targeting other overexpressed biomarkers responsible for tumor development or progression are also under investigation in gliomas, including RTKs [ephrin receptor A2 (EphA2) (395) and EGFR (396–400)], stem cell marker, CD133 (401), fibroblast activation factor-alpha (FAP-α) (402), Delta-like ligand 4 (Dll4, which is a ligand for a membrane receptor in different signaling pathways) (58), HGF (244), and MMP (403).

### Others

ImmunoPET studies on other malignancies are still in the preclinical setting. For instance, early murine studies targeting EGFR and CA 19-9 were promising in bladder cancer models (404, 405). An anti-CD3 probe also detected T-cell infiltration in bladder cancer-bearing mice, reflecting the potential for future studies on immunotherapy response prediction in urothelial cancers (406).

Evaluating mesothelioma, an anti-MSLN Ab, [^89^Zr]Zr-amatuximab, detected MSLN-expressing xenografts (308, 407), which can play a part in the patient selection for anti-MSLN targeted therapy. EGFR is also overexpressed in mesotheliomas (408). In this regard, receptor-specific uptake of [^86^Y]Y-cetuximab and [^86^Y]Y-panitumumab were documented, showing more favorable tumor-targeting characteristics with the latter (158). Also, a radiolabeled anti-EGFR Ab that selectively targets an epitope of EGFR detected EGFR-expressing xenografts (408). In cholangiocarcinoma cell lines, L1CAM overexpression, a cell adhesion molecule, was depicted using [^64^Cu]Cu-cA10-A3 probe (76, 409, 410). Recently, the role of receptor activator of nuclear factor-kappa B/ligand (RANK/RANKL) is recognized in resistance to immunotherapy. Its expression was non-invasively assessed by [^89^Zr]Zr-denosumab in cervical and HNSCC xenografts (90). Finally, CD44v6 overexpression was documented in vulva cancer cell lines using an anti-CD44v6 minibody, using [^124^I]I-AbD19384 (271).

## Conclusion

In the era of precision medicine and molecular targeted therapy, the need for accurate targeted imaging is inevitable. Given the inherent favorable characteristics, immunoPET seems very promising in this regard. A broad spectrum of both tumor-specific and common general molecules in different malignancies has already become targets for radioimmunoimaging of cancer. However, only a few have been introduced in clinical studies (Supplementary Table 1). Some common obstacles to the wide implementation of immunoPET include the high costs, advanced technology to commercially produce pure mAbs, difficulties in conjugation and in vivo tracer stability, as well as high circulation time and physiologic uptake after administration. Recent developments are answering these needs (97, 411) and will continue to evolve. Although immunoPET can become a diagnostic tool in specific conditions, its primary role seems to be a complementary imaging tool for therapy guidance.

Almost all studies mention the heterogeneous uptake of probes in different tumors. That is where the immunoPET strength lies, i.e., non-invasively depicting the heterogeneity of tumoral lesions. ImmunoPET tracks target expression and pharmacokinetics of mAbs in vivo before and after certain treatments, pointing out its potential value for patient recruiting and response monitoring of targeted therapies. Noteworthy, smaller Ab fragments provide more favorable imaging properties that can help increase the detection rate and accuracy of the imaging. However, the implementation of whole Abs is crucial since these structures are used for treatment, and the goal is to demonstrate their in vivo distribution.

Another advantage of developing new radioimmunoimaging probes is the theranostic application. The probes can be labeled with positron-emitting imaging and beta/alpha-emitting therapeutic radiometals, providing another possible treatment option for different malignancies.

Besides developing new targeting probes, future studies should also focus on the predictive and prognostic value of the radioimmunotracers after targeted therapies to further elaborate on their impact on treatment selection.

## Author Contributions

RM-F, CP, and MB came up with the main study topic and designed the outline. RM-F and BA performed the initial search and screened the literature to finalize the topics to cover. RM-F, BA, SR, ZA, and MM performed the literature search and extracted and summarized the data. All authors contributed to writing the initial draft of the manuscript under the supervision of RM-F, CP, and MB. ZA, BA, and RM-F drafted the tables. RM-F and SR prepared the figure. BA proofread and edited the final version of the manuscript. All authors read and approved the final version prior to submission.

## Conflict of Interest

The authors declare that the research was conducted in the absence of any commercial or financial relationships that could be construed as a potential conflict of interest.

## Publisher's Note

All claims expressed in this article are solely those of the authors and do not necessarily represent those of their affiliated organizations, or those of the publisher, the editors and the reviewers. Any product that may be evaluated in this article, or claim that may be made by its manufacturer, is not guaranteed or endorsed by the publisher.

## Glossary

[^18^F]FDG: [^18^F]FluoroDeoxyGlucose
[^18^F]FDOPA: [^18^F]Fluoro-L-Dopa
ACKR3: Atypical Chemokine Receptor 3
ADC: Antibody-Drug Conjugate
ATC: Anaplastic Thyroid Carcinoma
BBB: Blood-Brain Barrier
bsAb: bispecific Antibody
CA 15-3: Cancer Antigen 15-3
CA 19-9: Carbohydrate Antigen 19-9
CA125: Carbohydrate Antigen 125
CA6: Carbonic Anhydrase 6
CA-IX: Carbonic Anhydrase-IX
CD: Clusters of Differentiation
CDCP1: CUB domain-containing protein 1
CEA: Carcinoembryonic Antigen
CH: Constant Heavy Chain
CNS: Central Nervous System
CT: Computed Tomography
CTLA-4: Cytotoxic T-lymphocyte Associated Protein
CXCR: C-X-C Chemokine Receptor
Df: Desferrioxamine
Dll4: Delta-like ligand 4
dsFv: disulfide Fragment variable
ECM: Extracellular Matrix
EGFR: Epidermal Growth Factor Receptor
EphA2: Ephrin Receptor A2
Fab: antigen-binding Fragment
FAP-α: Fibroblast Activation Factor-Alpha
Fc: crystallizable Fragment
Gal-3: β-galactoside-binding protein galectin-3;
GITR: Glucocorticoid-Induced Tumor Necrosis Factor Receptor
GPC3: Glypican 3
GRP78: Glucose-regulated protein 78
HCC: Hepatocellular Carcinoma
HER: Human Epithelial Growth Factor Receptor;
HGF: Hepatocyte Growth Factor
HNSCC: Head and Neck Squamous Carcinoma
HSG: Histamine-Succinyl-Glycine
HSP90: Heat Shock Protein 90
ICAM-1: Intercellular Adhesion Molecule-1
ICI: Immune Checkpoint Inhibitor
ICOS: Inducible T-Cell Costimulatory Receptor
IGF-1R: Insulin-like Growth Factor-1 Receptor
IgG: Immunoglobulin G
IHC: Immunohistochemistry
KRAS: Kirsten Rat Sarcoma Virus
LGR5: Leucine-rich repeat-containing G-protein coupled Receptor 5
mAb: monoclonal Antibody
MMP: Matrix metalloproteinase
MRI: Magnetic Resonance Imaging
MSLN: Mesothelin
MTC: Medullary Thyroid Carcinoma
mTOR: Mammalian Target of Rapamycin
MUC: Mucin
NIRF: Near-Infrared Fluorophore
NSCLC: Non-Small Cell Lung Cancer
OX40: Tumor Necrosis Factor (Ligand) Superfamily, Member 4
PD-1/PD-L1: Programmed Cell Death Protein-1/Ligand
PDAC: Pancreatic Ductal AdenoCarcinoma
PDGF/PDGFR: Platelet-Derived Growth Factor/Receptor
PET: Positron Emission Tomography
PSCA: Prostate Stem Cell Antigen
PSMA: Prostate Specific Membrane Antigen
PTC: Papillary Thyroid Carcinoma
RAGE: Receptor for advanced glycation end products
RANK/RANKL: Receptor Activator of Nuclear Factor-Kappa B/Ligand
RCC: Renal Cell Carcinoma
RTK: Receptor Tyrosine Kinase
scdsFv: single-chain disulfide Fragment variable
scFv: single-chain Fragment variable
sdAb: single-domain Antibody
SUV: Standardized Uptake Value
TAG-72: Tumor-Associated Glycoprotein-72
TAM: Tumor-Associated Macrophage
TBR: Target-to-Background Ratio
TF: Tissue factor
TF: Tissue Factor (CD142)
TfR: Transferrin receptor
TGF: Transforming Growth Factor
TIL: Tumor Infiltrating Lymphocyte
TIM-3: T-Cell Immunoglobulin and Mucin Domain-Containing-3
TNF: Tumor Necrosis Factor
TROP-2: Trophoblast cell-surface antigen-2, known as tumor-associated calcium signal transducer 2
VEGF/VEGFR: Vascular Endothelial-Derived Growth Factor/Receptor.
