# Supplementary material for: ImmunoPET: Antibody-Based PET Imaging in Solid Tumors
Source: Front Med (Lausanne). 2022 Jun 28;9:916693. doi: 10.3389/fmed.2022.916693 (PMC9273828; doi:10.3389/fmed.2022.916693)
Supplement: Supplementary file 1 [file Table_1.docx]

| Supplementary table 1. clinical stage ImmunoPET imaging probes | | | | | | |
| --- | --- | --- | --- | --- | --- | --- |
| Author | Year | Type of cancer | Radiotracer | Target molecule | Targeting moiety | Results |
| [van Loon](https://pubmed.ncbi.nlm.nih.gov/?term=van+Loon+J&cauthor_id=28012793) (1) | 2017 | Head and neck and lung cancer | [^89^Zr]Zr- cetuximab | EGFR | IgG1 mAb | No toxicity emerged after administration.  In all but one patient, TBR>1 was noted.  Max TBR was:4.56. |
| [Bahce](https://pubmed.ncbi.nlm.nih.gov/?term=Bahce+I&cauthor_id=26055936) (2) | 2014 | Non-small cell lung cancer | [^89^Zr]Zr-bevacizumab | VEGF-A | IgG1 mAb | [89Zr]Zr-bevacizumab tumoral and metastases’ uptake was approximately 4 times higher than in the tumor free tissues (healthy muscle, lung, and fat). |
| [Niemeijer](https://pubmed.ncbi.nlm.nih.gov/?term=Niemeijer+AN&cauthor_id=30405135) (3) | 2018 | Non-small cell lung cancer | [^89^Zr]Zr-nivolumab  [^18^F]F-BMS-986192 | PD-1  and PD-L1 | IgG4 mAb | No adverse events higher than grade 3 were detected.  High tracer uptake was noted in the spleen, lymphocytes, dendritic cells, and the liver for both tracers.  Both tracers had adequate tumor to BG uptake.  Uptake heterogeneity between patients and lesions was noted.  Tracer uptake was correlated with IHC.  Tracer uptake was related to partial response. |
| [Niemeijer](https://pubmed.ncbi.nlm.nih.gov/?term=Niemeijer+AN&cauthor_id=30405135) (4) | 2022 | Non-small cell lung cancer | [^89^Zr]Zr-pembrolizumab | PD-1 | IgG4 | Tracer uptake was seen in the blood pool, liver and spleen.  Uptake heterogeneity between patients and lesions was noted.  47.2% of tumor lesions with ≥20 mm long axis diameter showed tracer uptake.  No significant correlation with IHC was noted. |
| [Pandit-Taskar](https://pubmed.ncbi.nlm.nih.gov/?term=Pandit-Taskar+N&cauthor_id=26175541) et al.(5) | 2015 | Prostate cancer | [^89^Zr]Zr-DFO-huJ591 | extracellular domain of PSMA | Antibody | overall accuracy of [^89^Zr]Zr-DFO-huJ591:   1. Osseous lesions: 95.2% (20 of 21) 2. Soft-tissue lesions: 60% (15 of 25) |
| O['Donoghue](https://pubmed.ncbi.nlm.nih.gov/?term=O%27Donoghue+JA&cauthor_id=28637800) et al. (6) | 2018 | Esophagogastric adenocarcinoma | [^89^Zr]Zr-trastuzumab | HER2 | Antibody | 1. No significant toxicity 2. plasma volume: 102% (range, 78%-113%) of injected dose 3. T_1/2β:_ 111 h (range, 78-193 h) 4. No to minimal uptake in 3 patients and median of 6.8 in 7 patients. 5. Target organ: Liver and heart wall |
| [Sanchez-Vega](https://pubmed.ncbi.nlm.nih.gov/?term=Sanchez-Vega+F&cauthor_id=30463996) et al.(7) | 2018 | ERBB2-Amplified esophagogastric cancer | [^89^Zr]Zr-trastuzumab | HER2 | Antibody | 1. Median pre-treatment SUVmax was 5.6 (range, 6.4 to 23.8). 2. Four patients (50%): no tracer uptake of metastases on PET, all progressed within 6 weeks. 3. Three patients: high uniform tracer uptake on PET, all regressed. |
| Kok et al.(8) | 2022 | Melanoma and non-small-cell lung cancer | [^89^Zr]Zr-pembrolizumab | PD-1 | humanized monoclonal IgG4 antibody | [^89^Zr]Zr-pembrolizumab uptake correlated with:   1. tumor response (P = 0.014) 2. progression-free (P = 0.0025) 3. overall survival (P = 0.026). |
| Dijkers et al. (9) | 2010 | breast cancer | [^89^Zr]Zr-trastuzumab | HER2 | Antibody | PET imaging of HER2-positive lesions in patients with metastatic breast cancer using [^89^Zr]Zr-trastuzumab indicated:   1. Four to five days after injection is the best time to evaluate tracer uptake by tumors. 2. Optimal dose of [^89^Zr]Zr-trastuzumab is 50 mg for trastuzumab-naive patients and 10 mg for patients treated with trastuzumab. 3. Visibility of most known lesions and some undetected lesions. 4. Higher relative uptake values (RUVs) in liver, bone and brain lesions compared to normal liver, spleen, kidney and brain tissue. |
| Ulaner et al.(10) | 2018 | breast cancer | [^89^Zr]Zr-Pertuzumab | HER2 | Antibody | Safety evaluation and dosimetry of [^89^Zr]Zr-Pertuzumab PET/CT presented:   1. No toxicities. 2. The organs with the highest doses, including the liver, kidneys, and heart wall. 3. Average effective dose of 0.54 ± 0.07 mSv/MBq. 4. Optimal imaging time of 5-8 days after administration. 5. Successful HER2-targeted imaging at multiple sites of malignancy. |
| Laforest et al.(11) | 2016 | breast cancer | [^89^Zr]Zr-trastuzumab | HER2 | Antibody | Evaluation of safety, human radiation dosimetry, and optimal imaging time of [^89^Zr]Zr-trastuzumab in patients with HER2-positive breast cancer identified:   1. High-quality images. 2. The highest tumor-to-nontumor ratio was 5 ± 1 days post-injection. 3. The liver was the dose-limiting organ. 4. The effective dose was 0.47 mSv/MBq. 5. No adverse effects observed. |
| Tamura et al.(12) | 2013 | breast cancer | [^64^Cu]Cu-DOTA-trastuzumab | HER2 | Antibody | Evaluation of the safety, distribution, and internal dosimetry of [^64^Cu]Cu-DOTA-trastuzumab indicated:  1. Feasibility of [^64^Cu]Cu-DOTA-trastuzumab PET to identify HER2-positive lesions in patients with primary and metastatic breast cancer.  2.The best time for imaging was 48 hours after injection.  3. High blood pool but low uptake in normal tissue.  4. Acceptable dosimetry at the dose required for adequate PET imaging. |
| Keyaerts et al.(13) | 2016 | breast cancer | [^68^Ga]Ga-HER2-Nanobody | HER2 | Nanobody | Phase I study, assessment of safety, biodistribution, and dosimetry as well as investigation of tumor-targeting potential of [^68^Ga]Ga-HER2-Nanobody PET/CT resulted:   1. No adverse reactions and dosimetry comparable to other routinely used PET tracers (effective dose of 0.043 mSv/MBq), and the urinary bladder wall as the critical organ with a dose of 0.406 mGy/MBq. 2. Rapid blood clearance (blood pool activity 10% of the injected dose 1 hour after injection), favorable biodistribution with highest uptake in kidneys, liver and intestine and very low background levels in all other organs. 3. Tracer accumulation above background levels in most identified metastatic disease sites, but more variable in primary lesions. |
| Beylergil et al.(14) | 2013 | breast cancer | [^68^Ga]Ga-DOTA-F(ab')2-trastuzumab | HER2 | F(ab′)2 | Pilot study of the toxicity, pharmacokinetics, biodistribution, and dosimetry of [^68^Ga]Ga-DOTA-F(ab')2-trastuzumab in HER2-negative and HER2-positive breast cancer demonstrated:   1. The safety of the agent, with a T½ of ≈ 3.6 ± 0.9 h. 2. The critical organ was the kidney (mean dose of 0.383 cGy/37 MBq). 3. Tumor targeting was seen in 4 out of 8 patients with HER2-positive disease. |
| Sandström et al.(15) | 2016 | breast cancer | [^68^Ga]Ga-ABY-025 | HER2 | Affibody | Measurement of biodistribution and estimation of radiation dosimetry of [^68^Ga]Ga-ABY-025 for 2 different peptide mass doses in a single group of patients with metastatic breast cancer presented:  1. The organs with the highest absorbed dose are the kidneys, followed by the liver, slightly but significantly higher for LD.  2. The effective dose for a typical 200-MBq administration of [^68^Ga]Ga-ABY-025, 6.0 mSv for LD and 5.6 mSv for HD, is slightly higher than for other [^68^Ga]Ga-labeled tracers, mainly due to higher uptake in the liver and the kidneys. |
| Velikyan et al.(16) | 2019 | breast cancer | [^68^Ga]Ga-ABY-025 | HER2 | Affibody | Testing of the fully automated [^68^Ga]Ga-ABY-025:  1. Radiochemical purity of 98.7 ± 0.6% with a total peptide content of 315 ± 15 µg (n = 3).  2. Acceptable purity, sterility, endotoxin content, residual solvent content and sterile filter integrity.  3. Stable at room temperature for at least 2 hours.  4.Detection of primary tumor and metastases (SUVmax of 8.3 and 16.0, respectively) with similar observed biodistribution in the Phase I study.  5. Validation of automated production of [^68^Ga]Ga-ABY-025 for use in standardized multicenter phase II and III clinical trials and routine clinical use. |
| Mortimer et al.(17) | 2014 | breast cancer | [^64^Cu]Cu-DOTA-trastuzumab | HER2 | Antibody | Evaluation of [^64^Cu]Cu-DOTA-trastuzumab PET/CT to detect and measure tumor uptake of trastuzumab in patients with HER2-positive metastatic breast cancer:  1. Pretreatment with 45 mg trastuzumab decreased liver uptake of ^64^Cu (by approximately 75%), with no significant effect on tumor uptake.  2. High sensitivity in HER2-positive metastatic breast cancer (77% and 89% for day 1 and day 2, respectively) and effective in monitoring disseminated disease.  3. No toxicities. |
| Ulaner et al.(18) | 2017 | breast cancer | [^89^Zr]Zr-trastuzumab | HER2 | Antibody | Evaluation of [^89^Zr]Zr-trastuzumab PET/CT of HER2-positive metastases in patients with HER2-negative primary breast cancer indicates that it is effective in detecting unexpected HER2-positive metastases from HER2-negative primary breast cancer, although false-positive Zr-trastuzumab-avid foci pose a challenge to the application of this tracer. |
| Bensch et al.(19) | 2018 | breast cancer  bladder cancer | [^89^Zr]Zr- atezolizumab | PD-L1 | Antibody | First in human study to evaluate the feasibility of imaging with [^89^Zr]Zr- atezolizumab (anti-PD-L1):  1. High tracer uptake in lymphoid tissue and at sites of inflammation.  2. High but heterogeneous tumor uptake (varying within and between lesions, patients, and tumor types).  3. Pre-treatment PET signal being a better predictor of response to atezolizumab treatment than IHC or RNA sequencing-based predictive biomarkers. |
| Rousseau et al.(20) | 2020 | breast cancer | Pretargeting with anti-CEA/anti- IMP288 bispecific antibody, followed by [^68^Ga] Ga-IMP288 | CEA | Bispecific antibody | Comparison of a bispecific anti-CEA antibody immunoPET with [18F]F- FDG-PET, CT and MRI in HER-2 negative, CEA positive metastatic breast cancer patients showed:   1. Immuno-PET had a higher sensitivity than CT or [18F]F- FDG-PET in lymph nodes (92.4% vs. 69.7% and 89.4%, respectively) and liver metastases (97.3% vs. 92.1% and 94.8%, respectively), whereas sensitivity was lower for lung metastases (48.3% vs. 100% and 75.9%, respectively). 2. Immuno-PET showed higher sensitivity than MRI or [18F]F- FDG PET for bone lesions (95.8% vs. 90.7% and 89.3%, respectively). 3. In contrast to [18F]F- FDG PET, immuno-PET disclosed brain metastases. 4. Despite equivalent tumor SUVmax, SUVmean, and total tumor volume, total lesion activity was significantly higher with immuno-PET than with [18F]F- FDG PET. |
| Gaykema et al.(21) | 2013 | breast cancer | [^89^Zr]Zr-bevacizumab | (VEGF)-A | Antibody | Evaluation of VEGF-A expression in primary breast cancer by [^89^Zr]Zr-bevacizumab PET showed:  1. Twenty-five of 26 breast tumors (mean size ± SD, 25.1 ± 19.8 mm; range, 4-80 mm) in 23 patients were visualized.  2. Higher SUV max values in tumors (1.85 ± 1.22; range, 0.52-5.64) compared with normal breasts (0.59 ± 0.37; range, 0.27-1.69; P < 0.001) confirmed the correlation of [^89^Zr]Zr-bevacizumab tumor uptake with histochemical VEGF-A tumor levels (r = 0.49)  3. Detection of 4 of 10 axillary lymph node metastases (SUV max, 2.66 ± 2.03; range, 1.32-5.68). |
| Menke-van et al.(22) | 2015 | colorectal cancer | [^89^Zr]Zr-cetuximab | EGFR | Antibody | Evaluation of biodistribution and tumor uptake of [^89^Zr]Zr-cetuximab PET/CT indicates tumor uptake, albeit the correlation between uptake and response to cetuximab warrants further clinical validation. |
| van Helden(23) | 2020 | colorectal cancer | [^89^Zr]Zr-cetuximab | EGFR | Antibody | Evaluation of [^89^Zr]Zr-cetuximab tumor accumulation as a biomarker potentially predictive and as a determinant of escalating dosing strategy:  1. No value of [^89^Zr]Zr-cetuximab tumor accumulation for predicting treatment response in patients with RAS wild-type mCRC undergoing cetuximab monotherapy.  2. BRAF mutations, right-sidedness, and low sEGFR correlate with intrinsic resistance to cetuximab. |
| Touchefeu et al.(24) | 2021 | colorectal cancer | Pretargeting with anti-CEA/anti- IMP288 bispecific antibody, followed by [^68^Ga] Ga-IMP288 | CEA | Bispecific antibody | To evaluate the imaging performance of pre-targeted immuno-PET using a recombinant anti-CEA-BsMAb, TF2 and the [^68^Ga]Ga-IMP288 in patients with mCRC:   1. safety and feasibility of pre-targeted immuno-PET using anti-CEA/anti-IMP288 BsMAb and [^68^Ga]Ga-IMP288 with promising diagnostic performance. 2. median SUV max, MTV and TLG 7.65 [3.98-13.94, SD 3.37], 8.63 cm3 [1.98-46.64; SD 14.83] and 37.90 cm3 [8.07-127.5; SD 43.47] for immuno-PET lesions, respectively. 3. in per-lesion analysis, sensitivity, specificity, positive predictive value, and negative predictive value were 82%, 25%, 82%, and 25%, respectively, for the combination of EUS/CT/MRI; 76%, 67%, 87%, and 33% for FDG-PET; and 88%, 100%, 100%, and 67% for immuno-PET. 4. Management implications in 2 patients. |
| Moek et al.(25) | 2019 | gastrointestinal adenocarcinoma | [^89^Zr]Zr-AMG 211 | CEA  CD3 | 55 kDa bispecific T-cell engager | Evaluation of AMG 211 biodistribution in patients with advanced gastrointestinal adenocarcinomas denoting:  1. High, specific [^89^Zr]Zr-AMG 211 uptake in CD3-rich lymphoid tissues.  2. Inter- and intra-individual heterogeneous tumor uptake.  3. Optimal imaging dose before AMG 211 treatment: 200 μg [^89^Zr]Zr-AMG 211 + 1,800 μg cold AMG 211. serum half-life of the tracer: 3.3 hours.  4.No visualization of tumor lesions during AMG 211 treatment, possibly due to target saturation.  5. Renal excretion |
| van Brummelen et al.(26) | 2018 | colorectal cancer | [^89^Zr]Zr-CEA-IL2v | CEA | immunocytokine | Evaluation of [^89^Zr]Zr-CEA-IL2v biodistribution and tumor accumulation showed:   1. Preferential uptake in CEA positive tumors (support for selective and targeted tumor accumulation). 2. Non-significant dose-dependent tumor accumulation (higher accumulation at doses ≥20 mg). 3. Dose- and CEA-independent biodistribution. 4. Major accumulation in lymphoid tissue (IL-2R binding). 5. Lower exposure and tumor accumulation (%ID/mL(peak) 57% lower) in cycle 4 compared to cycle 1 (peripheral immune cells expansion. 6. Support of the therapeutic concept of CEA-IL2v. |
| O'Donoghue et al.(27) | 2011 | colorectal cancer | [^124^I]I-huA33 | A33 | Antibody | Quantitative study of antibody-antigen interactions in tumors and normal tissues:   1. High uptake in regions with antigen-positive tumor cells (mean, ;0.05 %ID/g), and antigen-positive normal colon, with linear relationships. 2. Hypothesized that the optimal strategy for radioimmunotherapy based on the A33 system will be a multistep treatment with a short-range radionuclide. |
| Carrasquillo et al.(28) | 2011 | colorectal cancer | [^124^I]I-huA33 | A33 | Antibody | Evaluation of [^124^I]I-huA33 localization,biodistribution, and safety in patients with colorectal cancer:   1. Good localization of [^124^I]I-huA33 in colorectal cancer. 2. No significant toxicity. 3. Accuracy quantification by [^124^I]I-huA33 PET. 4. No advantage of hepatic arterial infusion over intravenous administration. 5. No clinically significant changes in blood clearance by IVIG administration. |
| Lohrmann et al.(29) | 2019 | pancreatic cancer | [^89^Zr]Zr-MVT-2163 | CA19-9 | Antibody | Evaluation of HuMab-5B1 (MVT-2163) immuno-PET to identify the source of CA19-9 elevation in patients with pancreatic cancer:   1. Well tolerated and without severe side effects. 2. Imaging of known primary tumors and metastases as well as previously undetected subcentimeter lymph nodes located in typical metastatic sites. 3. High and increasing uptake by tumor tissue over time, indicating potential therapeutic application of HuMab-5B1 labeled with beta-emitting radioisotopes. |
| Nakamoto et al.(30) | 2021 | pancreatic cancer | [^18^F]F-FP-R(0)1-MG-F2 | human integrin α(v)β(6) | Cysteine knot peptide | Evaluation of the safety, biodistribution, dosimetry, and lesion uptake of [^18^F]F-FP-R(0)1-MG-F2 in patients with pancreatic cancer:   1. A safe PET radiopharmaceutical with an effective dose comparable to other diagnostic agents (Effective dose: 2.538 × 10(-2) mSv/MBq). 2. 1 hour after injection: areas of high [^18^F]F-FP-R(0)1-MG-F2 3. uptake: pituitary, stomach, duodenum, kidneys, and bladder (average SUVmean: 9.7-14.5), intermediate uptake: normal pancreas (average SUVmean: 4.5), low uptake: lungs and liver (average SUVmean < 1.0). 4. Detection of all known pancreatic tumors. Failure to detect small peri-pancreatic subcentimeter metastatic lymph nodes. 5. Detection of confirmed distant metastases in lung, liver, and peritoneum. |
| Lamberts et al.(31) | 2016 | Pancreatic and Ovarian Cancer | [^89^Zr]Zr-MMOT0530A | MSLN | Antibody | To evaluate [^89^Zr]Zr-MMOT0530A tumor uptake, whole-body distribution, and the association between uptake, treatment response, and MSLN expression in patients with pancreatic and ovarian cancer before treatment with anti-mesothelin antibody-drug conjugates:   1. Feasibility of visualization of pancreatic and ovarian cancer lesions and antibody biodistribution by [^89^Zr]Zr-MMOT0530A-PET and correlation of tumor uptake with IHC. 2. Appropriate dose: 10 mg MMOT0530A and optimal imaging time 4 and 7 days after injection. 3. Mean SUVmax of tumor uptake of 13.1, 4 days post-injection, with 11.5 (±7.5) in (N = 17) pancreatic and 14.5 (±8.7) in (N = 20) ovarian cancer lesions. 4. A mean 2.4-fold (±1.10) difference in uptake between tumor lesions within patients. 5. Normal distribution in blood, liver, kidney, spleen and intestine. 6. Best response to DMOT4039A: partial response in one patient. |
| van Loon et al.(1) | 2017 | head and neck cancer  lung cancer | [^89^Zr]Zr-cetuximab | EGFR | Antibody | To determine the safety of [^89^Zr]Zr-cetuximab and evaluate tumor uptake:   1. [^89^Zr]Zr-cetuximab was safe. 2. Recommended dose: 60 MBq, with a minimum scanning interval of 6 days. 3. Tumor-to-blood ratio>1 was obtained in all except one patient, with a maximum of 4.56 with no differences between dosing schedules. |
| Börjesson et al.(32) | 2006 | head and neck cancer | [^89^Zr]Zr-U36 | CD44v6 | Antibody | To evaluate the diagnostic imaging performance of [^89^Zr]Zr-U36 in patients with HNSCC at high risk of lymph node metastases:   1. Detection of all primary tumors (n = 17) and LN metastases in 18 of 25 positive levels (sensitivity 72%) and in 11 of 15 positive sites (sensitivity 73%). 2. Correct interpretation in 112 of 121 operated levels (accuracy 93%) and in 19 of 25 operated sites (accuracy 76%). 3. For CT and MRI, sensitivities of 60% and 73% and accuracies of 90% and 80% per level and side, respectively, were observed. 4. Comparable performance to CT and MRI for detection of HNSCC LN metastases. |
| Divgi et al.(33) | 2013 | Renal cell carcinoma | [^124^I]I -girentuximab | CA-IX | Antibody | Comparison of sensitivity and specificity of [^124^I]I -girentuximab PET/CT and CECT:   1. Well-tolerated and feasible approach. 2. Average sensitivity: 86.2% (95% CI, 75.3% to 97.1%) for PET/CT and 75.5% (95% CI, 62.6% to 88.4%) for CECT (P = .023). 3. Average specificity: 85.9% (95% CI, 69.4% to 99.9%) for PET/CT and 46.8% (95% CI, 18.8% to 74.7%) for CECT (P = .005). 4. High inter- and intrareader agreement (κ-range, 0.87 to 0.92 for PET/CT; 0.67 to 0.76 for CECT; range, 87% to 100% for PET/CT; 73.7% to 91.3% for CECT, respectively). 5. Confirmation of the high accuracy of [^124^I]I -girentuximab PET/CT in noninvasive preoperative identification of the phenotype of ccRCC. |
| Divgi et al.(34) | 2007 | Renal cell carcinoma | [^124^I]I -girentuximab | CA-IX | Antibody | Use of iodine-[124I]I -girentuximab to characterize ccRCC:   1. Ability to accurately identify ccRCC with high negative predictive value for a less aggressive phenotype, leading to assisting in treatment decision-making. 2. Sensitivity 94% (95% CI 70-100%); negative predictive value 90% (55-100%), and specificity and positive predictive accuracy both 100% (66-100% and 78-100%, respectively). 3. The resulting high sensitivity and specificity support the potential clinical use of [^124^I]I-girentuximab in renal masses as an alternative to biopsy for lesion characterization. |
| Verhoeff et al.(35) | 2019 | Renal cell carcinoma | [^89^Zr]Zr-DFO-girentuximab | CA-IX | Antibody | Evaluating lesion detection by CECT, [^89^Zr]Zr-DFO-girentuximab PET/CT, and [^18^F]FDG PET/CT in detecting ccRCC lesions in patients with good or intermediate prognosis mccRCC:   1. Combination [^89^Zr]Zr-DFO-girentuximab PET/CT and CT identified more lesions than CT alone: 91% (95%CI: 87-94) vs 56% (95%CI: 50-62, p = 0.001), respectively, or more than CT and [^18^F]FDG-PET/CT combined (84% (95%CI: 79-88, p < 0.005)). 2. Identification of more bone and soft tissue lesions by PET/CT compared to CT alone. 3. Increase in lesion detection by adding [^89^Zr]Zr-DFO-girentuximab PET/CT and [^18^F]FDG PET/CT to CT compared to CT alone in newly diagnosed mccRCC patients with good and intermediate prognosis who are eligible for a wait-and-see approach. |
| Oosting et al.(36) | 2015 | Renal cell carcinoma | [^89^Zr]Zr -bevacizumab | VEGF-A | Antibody | Determination of tumor uptake of [^89^Zr]Zr -bevacizumab in mRCC patients before and during antiangiogenic treatment:   1. High uptake in mRCC, with notable heterogeneity between and within patients. 2. Visualization 125 evaluable tumor lesions in 22 patients, with a median SUVmax of 6.9 (range, 2.3-46.9). 3. Bevacizumab/interferon-α produced a median change in tumor SUVmax of -47.0% (range, -84.7% to +20.0%; P < 0.0001) at 2 weeks and another of -9.7% (range, -44.8% to +38.9%; P = 0.015) at 6 weeks. 4. In the sunitinib group, the mean change in tumor SUVmax at 2 weeks was -14.3% (range, -80.4 to +269.9; P = 0.006), but at 6 weeks, the mean change in tumor SUVmax was +72.6% (range, -46.4 to +236%; P < 0.0001) above baseline. 5. SUVmax was not related to plasma VEGF-A at all time points examined. 6. A high baseline SUVmax (>10.0 in the 3 most intense lesions) was associated with a longer time to progression (89.7 vs 23.0 weeks; hazard ratio, 0.22; 95% confidence interval, 0.05-1.00). |
| Merkx et al.(37) | 2021 | Renal cell carcinoma | [^89^Zr]Zr-DFO-girentuximab | CA-IX | Antibody | Assessment of safety, biodistribution and radiation dosimetry for [^89^Zr]Zr-DFO-girentuximab in patients with renal cell carcinoma:   1. No adverse events ≥ grade 3. 2. Successful differentiation between ccRCC and non-ccRCC lesions in all patients confirmed with histological data. 3. Organs with the highest doses (mean ± SD): liver (1.86 ± 0.40 mGy/MBq), kidneys (1.50 ± 0.22 mGy/MBq) and the heart wall (1.45 ± 0.19 mGy/MBq) 4. Mean whole body effective dose of 0.57 ± 0.08 mSv/MBq. 5. Median tumour-absorbed dose of 4.03 mGy/MBq (range 1.90-11.6 mGy/MBq). |
| Jansen et al.(38) | 2017 | Diffuse Intrinsic Pontine Glioma | [89Zr]Zr-Bevacizumab | VEGF-A | Antibody | To investigate tumor availability of bevacizumab, safety, and determine optimal timing for imaging in children with diffuse intrinsic pontine glioma (DIPG) by measuring tumor uptake of [^89^Zr]Zr-labeled bevacizumab by PET:   1. Safe and feasible. 2. Optimal time point for scanning: 144 hours after injection. 3. Focal uptake in 5/7 primary tumors (SUVs at 144 h post-injection were 1.0-6.7), but no significant uptake in normal brain. 4. Positive PET results in all metastases in 1 patient. 5. Inter- and intratumoral heterogeneity of uptake. 6. Predominant (in 4/5 patients) but variable uptake in contrast-enhanced MRI regions. 7. Mean effective dose: 0.9 mSv/MBq (SD, 0.3 mSv/MBq). |
| Matsuda et al.(39) | 2018 | Brain tumor | [^89^Zr]Zr-Df-IAB2M | PSMA | Minibody | To investigate the potential of PSMA for tumor neovasculature assessment of various brain tumors and the applicability of detection of PSMA expression in brain tumors by [^89^Zr]Zr-Df-IAB2M PET/CT:   1. Visualization of PSMA expression in high-grade gliomas and a metastatic brain tumor. 2. Positive correlation between the degree of uptake and the level of PSMA expression in tumor preparations. 3. High expression of PSMA in the vascular endothelium of high-grade gliomas and a metastatic brain tumor, but low expression in the vascular endothelium of PCNSL and radiation necrosis based on IHC results. |
| den Hollander et al.(40) | 2015 | High-Grade Glioma | [^89^ Zr]Zr-fresolimumab | TGF-β | Antibody | To investigate [89 Zr]Zr-fresolimumab uptake in recurrent high-grade gliomas and evaluate treatment outcomes in patients with recurrent high-grade gliomas treated with fresolimumab:   1. At day 4, tumor lesion SUVmax of 4.6 (range, 1.5-13.9) and normal brain tissue median SUV of 0.3 (range, 0.2-0.5). 2. Clinical or radiologic progression after 1-3 infusions of fresolimumab in all patients. 3. Median progression-free survival of 61 days (range, 25-80 days), 4. Median overall survival of 106 days (range, 37-417 days). |
| Bodet-Milin et al.(41) | 2016 | Medullary Thyroid Carcinoma | Pretargeting with anti-CEA/anti- IMP288 bispecific antibody, followed by [^68^Ga] Ga-IMP288 | CEA | Bispecific antibody | Optimization of molar doses and pretargeting interval of TF2 and ([^68^Ga] Ga-labeled IMP288 for immuno-PET in relapsed MTC patients in five cohorts (C1-C5) of 3 patients who received variable molar doses of TF2 and approximately 150 MBq (68)Ga-IMP288 after different pretargeting intervals (C1: 120 nmol TF2, 6 nmol IMP288, 24 h; C2: 120 nmol TF2, 6 nmol IMP288, 30 h; C3: 120 nmol TF2, 6 nmol IMP288, 42 h; C4: 120 nmol TF2, 3 nmol IMP288, 30 h; and C5: 60 nmol TF2, 3 nmol IMP288, 30 h):   1. In C1, T-SUVmax and T/MBP ranged from 4.09 to 8.93 and 1.39 to 3.72 at 60 min and 5.14 to 11.25 and 2.73 to 5.38 at 120 min, respectively. Because of the high MBP, the delay in C2 was increased to 30 hours, which increased T-SUV max and T/MBP. Further increasing the delay to 42 hours in C3 decreased T-SUV max and T/MBP, showing that 30 hours was the most favorable delay. In C4, the molar ratio of TF2 to peptide was increased to 40 (delay 30 h), resulting in a high T-SU Vmax but a higher MBP than in C2. In C5, the molar dose of TF2 was reduced, resulting in lower imaging performance. 2. Pharmacokinetics: rapid TF2 clearance, clear relationship between blood activity clearance and the ratio between the molar amount of injected peptide and the molar amount of circulating TF2 at the time of peptide injection. 3. Achieving high tumor uptake and contrast with targeted anti-CEA immuno-PET in MTC recurrent patients, especially using optimized pretargeting parameters: a BsMAb to peptide molar ratio of 20 and a 30-hour pretargeting delay. |
| Bodet-Milin et al.(42) | 2021 | Medullary Thyroid Carcinoma | Pretargeting with anti-CEA/anti- IMP288 bispecific antibody, followed by [^68^Ga] Ga-IMP288 | CEA | Bispecific antibody | To determine the sensitivity of immuno-PET in recurrent MTC patients compared with conventional imaging and [^18^F]F-DOPA PET/CT:   1. A total of 190 lesions were confirmed by IHC: 89 in LN, 14 in lung, 46 in liver, 37 in bone, and 4 in other sites (subcutaneous tissue, heart, brain, and pancreas). The number of abnormal foci detected by immuno-PET was 210 (174 (83%) true-positive). 2. A higher overall sensitivity for immuno-PET (92%) than [^18^F]F -DOPA PET/CT (65%). 3. Tumor SUVmax at 60 minutes ranged from 1.2 to 59.0, with values varying within and between patients. 4. For metastases, the higher sensitivity of immuno-PET than CT, [^18^F]F-DOPA PET/CT, or MRI for lymph nodes (98% vs. 83% for CT and 70% for [^18^F]F-DOPA PET/CT), liver (98% vs. 87% for CT, 65% for MRI). 87% for CT, 65% for [^18^F]F-DOPA PET/CT, and 89% for MRI) and bone (92% vs. 64% for [^18^F]F-DOPA PET/CT and 86% for MRI), and lower sensitivity for lung metastases (29% vs. 100% for CT and 14% for [^18^F]F-DOPA PET/CT). |
| Natarajan et al.(43) | 2020 | ovarian cancer breast cancer | [^64^Cu]Cu -DOTA-BFab | CA6 | Fab | [^64^Cu]Cu -DOTA-BFab in two patients:  well tolerated with no side effects.  No significant tracer uptake in either patient.  IHC data: weak to moderate CA6 expression consistent with [^64^Cu]Cu -DOTA-BFab PET signals. |
| ADC: Antibody drug conjugate**;** BG: background; BsMAb: bispecific monoclonal antibody; CA6: Carbonic Anhydrase 6; CA 19-9; cancer antigen 19-9; CA-IX: Carbonic Anhydrase-IX; ccRCC: clear cell renal carcinoma; CD: Clusters of Differentiation; CEA: Carcinoembryonic Antigen; CECT: contrast-enhanced computed tomography; CT: Computed Tomography; EGFR: epidermal growth factor receptor; ERBB2: erythroblastic oncogene B; Fab: antigen-binding Fragment; [^18^F]FDG: [^18^F]FluoroDeoxyGlucose; [^18^F]F-DOPA: [^18^F]F-l-dihydroxyphenylalanine; HNSCC: Head and Neck Squamous Carcinoma; HER2: human epidermal growth factor receptor 2; HSG: antihistamine-succinyl-glycine; IgG: Immunoglobulin G; IHC: Immunohistochemistry; IVIG: Intravenous immune globulin; LN: lymph node; mAb: monoclonal Antibody; MBP: mediastinum blood-pool; mccRCC: metastatic clear cell renal carcinoma; MRI: Magnetic Resonance Imaging; MSLN: Mesothelin; MTV: metabolic total volume; PD-1/PD-L1: Programmed Cell Death Protein-1/Ligand; PET: Positron Emission Tomography; PCNSL: Primary central nervous system lymphoma; PSMA: Prostate Specific Membrane Antigen; SUV: Standardized Uptake Value; T/MBP: T-SUVmax-to-mediastinum blood-pool SUVmean ratios; TBR: Target-to-Background Ratio; TF: Tissue factor; TLG: total lesion glycolysis; T-SUVmax: Tumor maximal SUV; VEGF: Vascular Endothelial-Derived Growth Factor | | | | | | |

**References**

1. van Loon J, Even AJG, Aerts H, Öllers M, Hoebers F, van Elmpt W, et al. PET imaging of zirconium-89 labelled cetuximab: A phase I trial in patients with head and neck and lung cancer. Radiother Oncol. 2017;122(2):267-73.

2. Bahce I, Huisman MC, Verwer EE, Ooijevaar R, Boutkourt F, Vugts DJ, et al. Pilot study of (89)Zr-bevacizumab positron emission tomography in patients with advanced non-small cell lung cancer. EJNMMI Res. 2014;4(1):35.

3. Niemeijer AN, Leung D, Huisman MC, Bahce I, Hoekstra OS, van Dongen G, et al. Whole body PD-1 and PD-L1 positron emission tomography in patients with non-small-cell lung cancer. Nature communications. 2018;9(1):4664.

4. Niemeijer AN, Oprea-Lager DE, Huisman MC, Hoekstra OS, Boellaard R, de Wit-van der Veen BJ, et al. Study of (89)Zr-Pembrolizumab PET/CT in Patients With Advanced-Stage Non-Small Cell Lung Cancer. J Nucl Med. 2022;63(3):362-7.

5. Pandit-Taskar N, O'Donoghue JA, Ruan S, Lyashchenko SK, Carrasquillo JA, Heller G, et al. First-in-Human Imaging with 89Zr-Df-IAB2M Anti-PSMA Minibody in Patients with Metastatic Prostate Cancer: Pharmacokinetics, Biodistribution, Dosimetry, and Lesion Uptake. J Nucl Med. 2016;57(12):1858-64.

6. O'Donoghue JA, Lewis JS, Pandit-Taskar N, Fleming SE, Schöder H, Larson SM, et al. Pharmacokinetics, Biodistribution, and Radiation Dosimetry for (89)Zr-Trastuzumab in Patients with Esophagogastric Cancer. J Nucl Med. 2018;59(1):161-6.

7. Sanchez-Vega F, Hechtman JF, Castel P, Ku GY, Tuvy Y, Won H, et al. EGFR and MET Amplifications Determine Response to HER2 Inhibition in ERBB2-Amplified Esophagogastric Cancer. Cancer Discov. 2019;9(2):199-209.

8. Kok IC, Hooiveld JS, van de Donk PP, Giesen D, van der Veen EL, Lub-de Hooge MN, et al. (89)Zr-pembrolizumab imaging as a non-invasive approach to assess clinical response to PD-1 blockade in cancer. Ann Oncol. 2022;33(1):80-8.

9. Dijkers EC, Oude Munnink TH, Kosterink JG, Brouwers AH, Jager PL, de Jong JR, et al. Biodistribution of 89Zr-trastuzumab and PET imaging of HER2-positive lesions in patients with metastatic breast cancer. Clin Pharmacol Ther. 2010;87(5):586-92.

10. Ulaner GA, Lyashchenko SK, Riedl C, Ruan S, Zanzonico PB, Lake D, et al. First-in-Human Human Epidermal Growth Factor Receptor 2-Targeted Imaging Using (89)Zr-Pertuzumab PET/CT: Dosimetry and Clinical Application in Patients with Breast Cancer. J Nucl Med. 2018;59(6):900-6.

11. Laforest R, Lapi SE, Oyama R, Bose R, Tabchy A, Marquez-Nostra BV, et al. [(89)Zr]Trastuzumab: Evaluation of Radiation Dosimetry, Safety, and Optimal Imaging Parameters in Women with HER2-Positive Breast Cancer. Mol Imaging Biol. 2016;18(6):952-9.

12. Tamura K, Kurihara H, Yonemori K, Tsuda H, Suzuki J, Kono Y, et al. 64Cu-DOTA-trastuzumab PET imaging in patients with HER2-positive breast cancer. J Nucl Med. 2013;54(11):1869-75.

13. Keyaerts M, Xavier C, Heemskerk J, Devoogdt N, Everaert H, Ackaert C, et al. Phase I Study of 68Ga-HER2-Nanobody for PET/CT Assessment of HER2 Expression in Breast Carcinoma. J Nucl Med. 2016;57(1):27-33.

14. Beylergil V, Morris PG, Smith-Jones PM, Modi S, Solit D, Hudis CA, et al. Pilot study of 68Ga-DOTA-F(ab')2-trastuzumab in patients with breast cancer. Nucl Med Commun. 2013;34(12):1157-65.

15. Sandström M, Lindskog K, Velikyan I, Wennborg A, Feldwisch J, Sandberg D, et al. Biodistribution and Radiation Dosimetry of the Anti-HER2 Affibody Molecule 68Ga-ABY-025 in Breast Cancer Patients. J Nucl Med. 2016;57(6):867-71.

16. Velikyan I, Schweighöfer P, Feldwisch J, Seemann J, Frejd FY, Lindman H, et al. Diagnostic HER2-binding radiopharmaceutical, [(68)Ga]Ga-ABY-025, for routine clinical use in breast cancer patients. Am J Nucl Med Mol Imaging. 2019;9(1):12-23.

17. Mortimer JE, Bading JR, Colcher DM, Conti PS, Frankel PH, Carroll MI, et al. Functional imaging of human epidermal growth factor receptor 2-positive metastatic breast cancer using (64)Cu-DOTA-trastuzumab PET. J Nucl Med. 2014;55(1):23-9.

18. Ulaner GA, Hyman DM, Lyashchenko SK, Lewis JS, Carrasquillo JA. 89Zr-Trastuzumab PET/CT for Detection of Human Epidermal Growth Factor Receptor 2-Positive Metastases in Patients With Human Epidermal Growth Factor Receptor 2-Negative Primary Breast Cancer. Clin Nucl Med. 2017;42(12):912-7.

19. Bensch F, van der Veen EL, Lub-de Hooge MN, Jorritsma-Smit A, Boellaard R, Kok IC, et al. (89)Zr-atezolizumab imaging as a non-invasive approach to assess clinical response to PD-L1 blockade in cancer. Nat Med. 2018;24(12):1852-8.

20. Rousseau C, Goldenberg DM, Colombie M, Sebille JC, Meingan P, Ferrer L, et al. Initial Clinical Results of a Novel Immuno-PET Theranostic Probe in Human Epidermal Growth Factor Receptor 2-Negative Breast Cancer. J Nucl Med. 2020;61(8):1205-11.

21. Gaykema SB, Brouwers AH, Lub-de Hooge MN, Pleijhuis RG, Timmer-Bosscha H, Pot L, et al. 89Zr-bevacizumab PET imaging in primary breast cancer. J Nucl Med. 2013;54(7):1014-8.

22. Menke-van der Houven van Oordt CW, Gootjes EC, Huisman MC, Vugts DJ, Roth C, Luik AM, et al. 89Zr-cetuximab PET imaging in patients with advanced colorectal cancer. Oncotarget. 2015;6(30):30384-93.

23. van Helden EJ, Elias SG, Gerritse SL, van Es SC, Boon E, Huisman MC, et al. [(89)Zr]Zr-cetuximab PET/CT as biomarker for cetuximab monotherapy in patients with RAS wild-type advanced colorectal cancer. Eur J Nucl Med Mol Imaging. 2020;47(4):849-59.

24. Touchefeu Y, Bailly C, Frampas E, Eugene T, Rousseau C, Bourgeois M, et al. Promising clinical performance of pretargeted immuno-PET with anti-CEA bispecific antibody and gallium-68-labelled IMP-288 peptide for imaging colorectal cancer metastases: a pilot study. Eur J Nucl Med Mol Imaging. 2021;48(3):874-82.

25. Moek KL, Waaijer SJH, Kok IC, Suurs FV, Brouwers AH, Menke-van der Houven van Oordt CW, et al. (89)Zr-labeled Bispecific T-cell Engager AMG 211 PET Shows AMG 211 Accumulation in CD3-rich Tissues and Clear, Heterogeneous Tumor Uptake. Clin Cancer Res. 2019;25(12):3517-27.

26. van Brummelen EMJ, Huisman MC, de Wit-van der Veen LJ, Nayak TK, Stokkel MPM, Mulder ER, et al. (89)Zr-labeled CEA-targeted IL-2 variant immunocytokine in patients with solid tumors: CEA-mediated tumor accumulation and role of IL-2 receptor-binding. Oncotarget. 2018;9(37):24737-49.

27. O'Donoghue JA, Smith-Jones PM, Humm JL, Ruan S, Pryma DA, Jungbluth AA, et al. 124I-huA33 antibody uptake is driven by A33 antigen concentration in tissues from colorectal cancer patients imaged by immuno-PET. J Nucl Med. 2011;52(12):1878-85.

28. Carrasquillo JA, Pandit-Taskar N, O'Donoghue JA, Humm JL, Zanzonico P, Smith-Jones PM, et al. (124)I-huA33 antibody PET of colorectal cancer. J Nucl Med. 2011;52(8):1173-80.

29. Lohrmann C, O'Reilly EM, O'Donoghue JA, Pandit-Taskar N, Carrasquillo JA, Lyashchenko SK, et al. Retooling a Blood-Based Biomarker: Phase I Assessment of the High-Affinity CA19-9 Antibody HuMab-5B1 for Immuno-PET Imaging of Pancreatic Cancer. Clin Cancer Res. 2019;25(23):7014-23.

30. Nakamoto R, Ferri V, Duan H, Hatami N, Goel M, Rosenberg J, et al. Pilot-phase PET/CT study targeting integrin α(v)β(6) in pancreatic cancer patients using the cystine-knot peptide-based (18)F-FP-R(0)1-MG-F2. Eur J Nucl Med Mol Imaging. 2021.

31. Lamberts LE, Menke-van der Houven van Oordt CW, ter Weele EJ, Bensch F, Smeenk MM, Voortman J, et al. ImmunoPET with Anti-Mesothelin Antibody in Patients with Pancreatic and Ovarian Cancer before Anti-Mesothelin Antibody-Drug Conjugate Treatment. Clin Cancer Res. 2016;22(7):1642-52.

32. Börjesson PK, Jauw YW, Boellaard R, de Bree R, Comans EF, Roos JC, et al. Performance of immuno-positron emission tomography with zirconium-89-labeled chimeric monoclonal antibody U36 in the detection of lymph node metastases in head and neck cancer patients. Clin Cancer Res. 2006;12(7 Pt 1):2133-40.

33. Divgi CR, Uzzo RG, Gatsonis C, Bartz R, Treutner S, Yu JQ, et al. Positron emission tomography/computed tomography identification of clear cell renal cell carcinoma: results from the REDECT trial. J Clin Oncol. 2013;31(2):187-94.

34. Divgi CR, Pandit-Taskar N, Jungbluth AA, Reuter VE, Gönen M, Ruan S, et al. Preoperative characterisation of clear-cell renal carcinoma using iodine-124-labelled antibody chimeric G250 (124I-cG250) and PET in patients with renal masses: a phase I trial. Lancet Oncol. 2007;8(4):304-10.

35. Verhoeff SR, van Es SC, Boon E, van Helden E, Angus L, Elias SG, et al. Lesion detection by [(89)Zr]Zr-DFO-girentuximab and [(18)F]FDG-PET/CT in patients with newly diagnosed metastatic renal cell carcinoma. Eur J Nucl Med Mol Imaging. 2019;46(9):1931-9.

36. Oosting SF, Brouwers AH, van Es SC, Nagengast WB, Oude Munnink TH, Lub-de Hooge MN, et al. 89Zr-bevacizumab PET visualizes heterogeneous tracer accumulation in tumor lesions of renal cell carcinoma patients and differential effects of antiangiogenic treatment. J Nucl Med. 2015;56(1):63-9.

37. Merkx RIJ, Lobeek D, Konijnenberg M, Jiménez-Franco LD, Kluge A, Oosterwijk E, et al. Phase I study to assess safety, biodistribution and radiation dosimetry for (89)Zr-girentuximab in patients with renal cell carcinoma. Eur J Nucl Med Mol Imaging. 2021;48(10):3277-85.

38. Jansen MH, Veldhuijzen van Zanten SEM, van Vuurden DG, Huisman MC, Vugts DJ, Hoekstra OS, et al. Molecular Drug Imaging: (89)Zr-Bevacizumab PET in Children with Diffuse Intrinsic Pontine Glioma. J Nucl Med. 2017;58(5):711-6.

39. Matsuda M, Ishikawa E, Yamamoto T, Hatano K, Joraku A, Iizumi Y, et al. Potential use of prostate specific membrane antigen (PSMA) for detecting the tumor neovasculature of brain tumors by PET imaging with (89)Zr-Df-IAB2M anti-PSMA minibody. J Neurooncol. 2018;138(3):581-9.

40. den Hollander MW, Bensch F, Glaudemans AW, Oude Munnink TH, Enting RH, den Dunnen WF, et al. TGF-β Antibody Uptake in Recurrent High-Grade Glioma Imaged with 89Zr-Fresolimumab PET. J Nucl Med. 2015;56(9):1310-4.

41. Bodet-Milin C, Faivre-Chauvet A, Carlier T, Rauscher A, Bourgeois M, Cerato E, et al. Immuno-PET Using Anticarcinoembryonic Antigen Bispecific Antibody and 68Ga-Labeled Peptide in Metastatic Medullary Thyroid Carcinoma: Clinical Optimization of the Pretargeting Parameters in a First-in-Human Trial. J Nucl Med. 2016;57(10):1505-11.

42. Bodet-Milin C, Faivre-Chauvet A, Carlier T, Ansquer C, Rauscher A, Frampas E, et al. Anti-CEA Pretargeted Immuno-PET Shows Higher Sensitivity Than DOPA PET/CT in Detecting Relapsing Metastatic Medullary Thyroid Carcinoma: Post Hoc Analysis of the iPET-MTC Study. J Nucl Med. 2021;62(9):1221-7.

43. Natarajan A, Srinivas SM, Azevedo C, Greene L, Bauchet AL, Jouannot E, et al. Two Patient Studies of a Companion Diagnostic Immuno-Positron Emission Tomography (PET) Tracer for Measuring Human CA6 Expression in Cancer for Antibody Drug Conjugate (ADC) Therapy. Mol Imaging. 2020;19:1536012120939398.
